# Eukaryote-to-eukaryote gene transfer gives rise to genome mosaicism in euglenids

**DOI:** 10.1186/1471-2148-11-105

**Published:** 2011-04-18

**Authors:** Shinichiro Maruyama, Toshinobu Suzaki, Andreas PM Weber, John M Archibald, Hisayoshi Nozaki

**Affiliations:** 1 Department of Biological Sciences, Graduate School of Science, University of Tokyo, 7-3-1 Hongo, Bunkyo, Tokyo 113-0033, Japan; 2 The Canadian Institute for Advanced Research, Program in Integrated Microbial Biodiversity, Department of Biochemistry and Molecular Biology, Dalhousie University, Sir Charles Tupper Medical Building, 5850 College Street, Halifax, Nova Scotia, B3H 1X5, Canada; 3 Department of Biology, Graduate School of Science, Kobe University, 1-1 Rokkodai-cho, Nada-ku, Kobe 657-8501, Japan; 4 Institute for Plant Biochemistry, Heinrich-Heine-University, Geb. 26.03.01, Universitätsstrasse 1, D-40225 Düsseldorf, Germany

## Abstract

**Background:**

Euglenophytes are a group of photosynthetic flagellates possessing a plastid derived from a green algal endosymbiont, which was incorporated into an ancestral host cell via secondary endosymbiosis. However, the impact of endosymbiosis on the euglenophyte nuclear genome is not fully understood due to its complex nature as a 'hybrid' of a non-photosynthetic host cell and a secondary endosymbiont.

**Results:**

We analyzed an EST dataset of the model euglenophyte *Euglena gracilis *using a gene mining program designed to detect laterally transferred genes. We found *E. gracilis *genes showing affinity not only with green algae, from which the secondary plastid in euglenophytes evolved, but also red algae and/or secondary algae containing red algal-derived plastids. Phylogenetic analyses of these 'red lineage' genes suggest that *E. gracilis *acquired at least 14 genes via eukaryote-to-eukaryote lateral gene transfer from algal sources other than the green algal endosymbiont that gave rise to its current plastid. We constructed an EST library of the aplastidic euglenid *Peranema trichophorum*, which is a eukaryovorous relative of euglenophytes, and also identified 'red lineage' genes in its genome.

**Conclusions:**

Our data show genome mosaicism in *E. gracilis *and *P. trichophorum*. One possible explanation for the presence of these genes in these organisms is that some or all of them were independently acquired by lateral gene transfer and contributed to the successful integration and functioning of the green algal endosymbiont as a secondary plastid. Alternative hypotheses include the presence of a phagocytosed alga as the single source of those genes, or a cryptic tertiary endosymbiont harboring secondary plastid of red algal origin, which the eukaryovorous ancestor of euglenophytes had acquired prior to the secondary endosymbiosis of a green alga.

## Background

Photosynthetic eukaryotes are distributed across multiple branches of the eukaryotic tree of life. Currently, six putative 'super-groups' of eukaryotes have been proposed: Opisthokonta, Amoebozoa, Rhizaria, Excavata, Chromalveolata, and Archaeplastida [1]. The origin of plastids (chloroplasts) from a cyanobacterial endosymbiont is referred to as primary endosymbiosis. Primary plastid-containing eukaryotes, namely green algae and land plants, glaucophytes and red algae, are classified into Archaeplastida, of which the monophyly is still debatable [2-7]. Subsequent to the evolution of primary plastids, two independent lineages of green algae were captured by two distinct lineages of phagotrophic protists via secondary (eukaryote-eukaryote) endosymbiosis, giving rise to the green secondary plastid-containing euglenophytes (Excavata) and chlorarachniophytes (Rhizaria). Chloroplast genome analyses suggest that the chlorarachniophyte plastid is derived from a green alga belonging to the ulvophyte-trebouxiophyte-chlorophyte group, while the ancestor of the euglenophyte plastid is related to prasinophyte green algae [8,9].

Red algae have also donated plastids to other eukaryotes by secondary endosymbiosis. It is well established that secondary plastids in many 'chromalveolate' taxa are derived from red algal endosymbiont(s), but the origin and evolutionary history of 'chromalveolate' plastids are more controversial than those of green algal ancestry [10-14]. Chromalveolata is composed of four major sub-groups (stramenopiles, alveolates, cryptophytes and haptophytes) and most sub-groups include non-photosynthetic members [1]. Recently, plastid-related genes and/or putative plastid-like organelles were found in several non-photosynthetic alveolate protists [15-19], and a novel photosynthetic lineage Chromerida was found and suggested to be a sister group of non-photosynthetic alveolates such as colpodellids [20,21]. Furthermore, phylogenomic analyses suggest that Chromalveolata may be a paraphyletic super-group; two major Chromalveolata lineages, stramenopiles and alveolates, are likely sister to another super-group, Rhizaria, to the exclusion of cryptophytes and haptophytes [22,23]. The recent description of *Roombia*, a new katablepharid, has led to a proposal to establish Hacrobia, a new taxonomic group that includes many protists formerly with uncertain taxonomical affiliations; katablepharids, (pico)biliphytes, centrohelids, telonemids and two traditional Chromalveolata lineages, cryptophytes and haptophtyes [24]. It was proposed that Hacrobia included multiple lineages that secondarily lost photosynthetic ability, although the existence of cryptic non-photosynthetic plastids in some hacrobian lineages, like those seen in some apicomplexans, cannot be ruled out. These data suggest that the history of plastid acquisition and loss in 'chromalveolates' is much more complicated than previously thought (reviewed in [5]).

Surprisingly, recent analyses of the nuclear genomes of the diatoms (stramenopiles; Chromalveolata) showed that thousands of diatom genes are similar to those of prasinophyte green algae, an observation that was interpreted as evidence for the existence of a cryptic endosymbiont of green algal origin in a 'chromalveolate' ancestor prior to the secondary endosymbiosis that gave rise to the extant secondary plastid of red algal origin [25]. These intriguing data still need to be investigated further, because the host nuclear phylogeny and the relationship between green algae and Chromalveolata is unclear and the host components of these two groups may be specifically related to one another [2,5].

The possible existence of multiple past endosymbioses is also debated in another super-group, the Excavata. Euglenophytes are photosynthetic flagellates belonging to the Euglenida (Excavata) [1]. The Euglenida include both heterotrophic and photoautotrophic protists, and share common ancestry with Kinetoplastea, which include the human parasites *Trypanosoma *and *Leishmania *[1]. Morphological, biochemical and phylogenetic analyses suggest that only the last common ancestor of the extant plastid-harboring euglenophytes experienced the secondary endosymbiosis, but not the common ancestor of Euglenozoa as a whole (Euglenida, Diplonemea and Kinetoplastea) [26]. However, the discovery of algal-type genes and the specific features of a mitochondrion-targeted protein in Kinetoplastea showing similarity to those of euglenophytes led to the hypothesis that a plastid was present in the common ancestor of Kinetoplastea, or Euglenozoa [27]. Previously, we developed an automated pipeline for single gene phylogenetic tree construction and found a number of genes showing cyanobacterial ancestry in the amoeboflagellate *Naegleria *(Heterolobosea, a sister group to Euglenozoa). One possible interpretation for the presence of these genes is that the primary endosymbiosis might have occurred in an ancestor of eukaryotes prior to the divergence of Excavata [28]. At any rate, among the extant Excavata lineages, the presence of plastids is thus far only known in euglenophytes [26].

A preliminary expressed sequence tag (EST) analysis of the model euglenophyte *Euglena gracilis *showed a complex history of nuclear genes in this organism [29], but many aspects of how the *E. gracilis *nuclear genome integrated genes from the green algal endosymbiont via secondary endosymbiosis are unclear. Moreover, recent molecular phylogenies suggested the presence of 'red lineage' genes in the nuclear genome of *E. gracilis*, but their origins and evolutionary histories have not been explored in detail [30-33]. Here we provide phylogenetic evidence for the presence of a number of genes of non-green algal origins in *E. gracilis *through an expanded EST survey using the laterally transferred gene mining pipeline [28]. We also discuss the possible evolutionary origins of these genes via lateral and/or endosymbiotic gene transfer (LGT/EGT).

## Results

To understand the nature and extent of genome mosaicism in *E. gracilis*, we searched for *E. gracilis *genes showing specific affinity to homologues of photosynthetic eukaryotes other than green algae, the latter being the unambiguous source of the endosymbiont that gave rise to the secondary plastid in euglenophytes. First, using the *E. gracilis *protein sequences generated from the EST database as queries, we assembled a set of sequences showing strong similarity to green algal/plant proteins in preliminary phylogenetic trees. We then identified the organisms to which the *E. gracilis *proteins showed the smallest distance to the query sequence on each tree. 528 and 621 *E. gracilis *queries were found to be 'closest' to the 'Viridiplantae' (green plants; namely green algae and land plants) and the 'red lineage' (i.e., red algae and secondary algae with plastids of red algal origin), respectively (Additional file 1: Supplementary table S1). We further checked the tree topologies of those putative 'red lineage'-like matches manually. We then chose the trees where the *E. gracilis *query was nested in, or specifically associated with, the 'red lineage' clade as a monophyletic group with high support values.

Finally we identified fourteen protein trees in which the *E. gracilis *sequence was monophyletic with the 'red lineage', not 'green' (Table 1). Among them, four trees included *E. gracilis *sequences placed in the Chromalveolata (plus Rhizaria) clade (CR clade) that is sister to red algal clades (CR+Red type), another two trees showed monophyletic clades of Chromalveolata sequences plus *E. gracilis *branching with green algae rather than red algae (CR+Green type). In the other eight trees, the euglenids are monophyletic with CR clades, but the sister group of the euglenids plus CR is unclear. Phylogenetic affiliations were sometimes not directly comparable due to differences in the availability of the gene/genome sequence data in each lineage. Nevertheless, the haptophytes (*Emiliania huxleyi*) and stramenopiles are associated with, and sister to, *E. gracilis *in most of the trees. Two 'red lineage' proteins closely related to the *E. gracilis *counterparts were found in the plastid-lacking euglenid *Peranema trichophorum*.

**Table 1:** 'Red lineage' genes in *E. gracilis*

Cluster ID	gene product	CR+Red	CR+Green	other	*Peranema *EST
0505	homogentisate phytyltransferase (HPT)	+			
1748	hypothetical protein	+			
5429	glucokinase	+			
7874	Clp protease proteolytic subunit (ClpP)	+			

2407	phosphoribulokinase (PRK)		+		
2525	folate-biopterin transporter (FBT)		+		

1468	zeaxanthin epoxidase (ZEP)			+	
2373	fructose 1,6-diphosphatase (FBP), plastidic			+	
2373	FBP, cytosolic			+	
4157	6-phosphogluconate dehydrogenase (GND)			+	+
4273	ADP/ATP transporter			+	+
5532	GTP-binding protein LepA			+	
6234	methionine adenosyltransferase (MAT)			+	
AAQ19605	fatty acid desaturase			+	

Original cluster IDs had "EEL0000" followed by the 4-digit numbers shown. The accession number is shown for the fatty acid desaturase gene, which was not found in the EST database.

### CR+Red type genes

**Figure 1:**
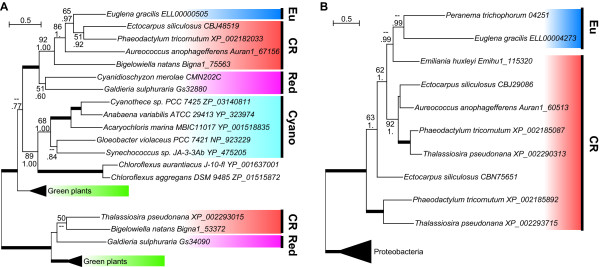
**Maximum likelihood (RAxML) tree of the 'red lineage' proteins found in euglenids**. The results of bootstrap analyses using RAxML (upper) and the Bayesian inference posterior probability values using MrBayes (lower) are shown on each branch. A, the best tree of the homogentisate phytyltransferase (HPT) family proteins shows that the *E. gracilis *HPT is closely related to the Chromalveolata and red algal homologues. B, the 'red lineage' genes encoding prokaryote-type ADP/ATP transporter have been found in euglenids and Chromalveolata. Thick branches represent BI and ML values not lower than 100 and 95, respectively. Eu, euglenids; CR, Chromalveolata plus Rhizaria; Red, red algae; Cyano, cyanobacteria. See supplementary figures for full trees.

We found that the gene encoding homogentisate phytyltransferase (HPT) from *E. gracilis *branches within the CR sub-clade, which itself is monophyletic with the red algal clade (Figure 1A, Additional file 2: Supplementary fig. S1A). These genes were included in the plastid and cyanobacteria type HPT gene family, and function in the vitamin E biosynthesis pathway [34]. Specific insertion/deletion sequences (Indels) shared with *E. gracilis *and the CR group supported the monophyly of these sequences. (Additional file 2: Supplementary fig. S1B)

*E. gracilis *was found to possess a gene encoding a hypothetical protein with a putative oxidoreductase domain. This protein family is thus far found only in photosynthetic organisms and conserved in the 'red lineage' including the cryptophyte nucleomorphs, which are derived nuclei of red algal origin. Green plant sequences were distributed on a branch separated from the CR+Red clade including the *E. gracilis *gene (Additional file 2: Supplementary fig. S2).

The glucokinase (EC 2.7.1.2) gene from *E. gracilis *was found to belong to the prokaryote-type gene family, which was also conserved among primary and secondary algae (Additional file 2: Supplementary fig. S3). Interestingly, animals, fungi, land plants and the excavates *Trypanosoma *and *Monocercomonoides *possess a different type of genes for this glucokinase enzyme [35,36], showing no sequence homology to the prokaryote-type genes. Although another excavate parasite *Giardia *possesses a prokaryote-type glucokinase [37], the *Giardia intestinalis *counterpart was sister to eubacteria, separate from the CR+Red clade in our preliminary analysis (data not shown). No land plant-like homologues were found in prasinophyte genomes.

In the *E. gracilis *EST database, we found a short fragment showing similarity to the ATP-dependent Clp protease proteolytic subunit (ClpP) (Additional file 2: Supplementary fig. S4A). The *E. gracilis *ClpP was more similar to nucleomorph-encoded cryptophyte homologues and the plastid-encoded green algal counterparts than to mitochondrial-localized ClpP homologues, suggesting that the *E. gracilis *ClpP may function in the plastid. Although this fragment is too short (299 bp) to allow construction of reliable phylogenetic trees, several characteristic amino acid residues are shared with cryptophyte nucleomorph-encoded proteins and those of pelagophytes (Additional file 2: Supplementary fig. S4B).

### CR+Green type genes

The plastid Calvin cycle enzyme phosphoribulokinase (PRK) is derived from the cyanobacterial ancestor of the organelle [30]. Molecular phylogenetic analysis of PRK genes suggested that extant Chromalveolata genes are related to their green algal counterparts, not red algae, and that these genes may not be derived from red algal endosymbionts engulfed in the ancestor of CR. Our phylogenetic analysis of PRK genes recovered the previously reported kinship [30] between green algal and Chromalveolata genes, and confirmed that the *E. gracilis *PRK nests within the Chromalveolata clade (Additional file 2: Supplementary fig. S5).

Folate/biopterin transporter (FBT) genes in CR have been shown to be most closely related to their counterparts in green plants [16,28]. In the FBT protein tree (Additional file 2: Supplementary fig. S6), the *E. gracilis *sequence is monophyletic with proteins from *Perkinsus *(Chromalveolata) and *Bigelowiella *(Rhizaria), and sister to the clade including the diatom *Thalassiosira *and prasinophyte genes. Separation of the *E. gracilis *gene from these prasinophyte genes is supported with the high support values (BI/ML = 1.00/100). In the CR assemblage and land plants, this gene family is highly duplicated and divergent, and the phylogenetic patterns are complicated. The tree topology suggests that both gene duplications in the ancestral lineages of CR and land plants, as well as more recent lineage specific duplications (or losses) have occurred. However, no evidence for gene duplication was found in red algae.

### *E. gracilis *genes with other affiliations

Zeaxanthin epoxidase (ZEP) is involved in the photoprotective xanthophyll cycle, catalyzing the addition of an epoxy group to zeaxanthin to form violaxanthin under low light conditions [32]. ZEP genes are not found in cyanobacteria and red algae thus far, and molecular phylogenetic studies suggested that ZEP genes in Chromalveolata might have been derived from prasinophytes via gene transfer events ([32] and Additional file 2: Supplementary fig. S7). Multiple duplicated ZEP genes are found in Chromalveolata genomes, forming various subclades (Additional file 2: Supplementary fig. S7). The *E. gracilis *gene was nested within a subclade of these genes, not affiliated with green plant gene clades.

Fructose-bisphosphatase (FBP) is a key glycolytic enzyme in eukaryotes and eubacteria. In photosynthetic eukaryotes, duplicated genes encoding FBP form another family functioning in carbohydrate metabolism such as the Calvin cycle in the plastid [31]. Teich et al. showed that both the plastid and cytosolic genes from *E. gracilis *were monophyletic with CR, not green plants [31]. We confirmed with detailed analysis that both types of *E. gracilis *sequences are nested within the CR clades in the plastid and cytosolic FBP gene families (Additional file 2: Supplementary fig. S8 and 9, respectively). However, the plastid FBP genes were duplicated in several lineages and the basal part of the CR FBP clade was not sufficiently resolved to verify the sister group to the *E. gracilis *plus CR clades.

The phylogenetic tree of 6-phosphogluconate dehydrogenase (GND) of cyanobacterial ancestry shows that the euglenid GNDs are monophyletic with the CR assemblage (Additional file 2: Supplementary fig. S10). A GND protein from the non-photosynthetic euglenid *P. trichophorum *is monophyletic with the *E. gracilis *counterpart and contained within the euglenid-CR clade. Thus the GND gene is likely a synapomorphic character of euglenophytes and non-photosynthetic euglenids [28,38]. We could not find a N-terminal extension on the *E. gracilis *homolog compared to cyanobacterial homologs (data not shown), thus it is likely that this enzyme functions in cytosol in this organism.

We found a 'red lineage' gene encoding a conserved protein with an ADP/ATP transporter domain in *E. gracilis *and *P. trichophorum *(Figure 1B, Additional file 2: Supplementary fig. S11). This gene was found only in euglenids and Chromalveolata among eukaryotes, in addition to several lineages of eubacteria. Stramenopiles possess duplicated forms of this gene.

We found another 'red lineage' gene, LepA, in *E. gracilis*. The LepA protein, also known as elongation factor 4 (EF4), is a GTP-binding protein conserved among eubacteria and eukaryotes. A previous study [39] showed that eukaryotic LepA genes are distributed in two clades, a mitochondrial LepA clade and a plastid one, with the plastid clade genes being sister to cyanobacterial homologues, suggesting that they are likely derived from the cyanobacterial ancestor of the plastid via EGT. Although *Escherichia coli *LepA was shown to catalyze one-codon backward movement of ribosome complexes *in vitro *[39], it has no apparent effect *in vivo *on the fidelity control of protein synthesis, rather presumably playing some role in protein folding [40]. In this study, the *E. gracilis *protein was nested in the CR subclade (BI/ML = 1.00/100) in the plastid clade (Additional file 2: Supplementary fig. S12).

Methionine adenosyltransferase (MAT) catalyzes the synthesis of *S*-adenosylmethionine, which is the major methyl donor and used as a substrate in a variety of methylation reactions. Genes encoding a divergent form of MAT, termed MATX, have been found in euglenophytes and CR, and it is suggested that the MATX genes were acquired via EGT [33]. Although the *E. gracilis *MATX is monophyletic with Chromalveolata MATX counterparts with high support values (BI/ML = 1.00/100), the origin of the MATX gene family is still unclear [33,41] (Additional file 2: Supplementary fig. S13).

Tripodi et al. [42] showed that the gene encoding Δ4 fatty acid desaturase in *E. gracilis *was closely related to the homologues of *Thalassiosira *and the labyrinthulid *Thraustochytrium *(stramenopiles). Although the sequence of the *E. gracilis *desaturase gene was not found in the EST database, we extended the phylogenetic analysis of this gene/protein with currently available data from other taxa. Our results (Additional file 2: Supplementary fig. S14A) are consistent with the previous study [42]. Examination of the protein sequence alignment revealed the presence of indels specific to the *Euglena*/*Thalassiosira *plus *Thraustochytrium *clade that are not shared with other excavate proteins from *Trypanosoma, Leishmania *and *Naegleria *(Additional file 2: Supplementary fig. S14B).

## Discussion

### Evolutionary history of the 'red lineage' genes in euglenophytes

We have shown that a number of *E. gracilis *genes are monophyletic with 'red lineage' genes and that some of them are nested within the CR clade, suggesting that the *E. gracilis *genes were acquired from algae in this group via LGT. In addition to the fact that no reports show a close phylogenetic relationship between the host cell components of euglenids and Chromalveolata (plus Rhizaria), the apparent absence of homologues of these *E. gracilis *genes in the available genome sequences of close relatives such as Kinetoplastea and the heterolobosean amoeboflagellate *N. gruberi *further suggest that the gene transfer events occurred in an ancestral lineage of euglenids, rather than euglenozoans as a whole.

**Figure 2:**
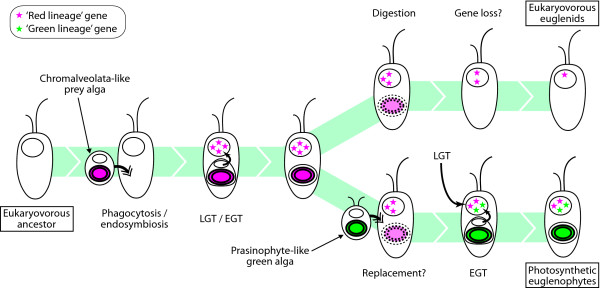
**Hypothesized evolutionary history of the 'red lineage' genes in euglenids**. In this model, the 'red lineage' genes have been acquired by the common ancestor of euglenophytes and eukaryovorous euglenids.

If the LGT scenario discussed above is true, what kind of eukaryote-to-eukaryote LGT could have occurred? One possible and most likely scenario is LGT from prey to predator, as suggested in an EST-based analysis of the mixotrophic chlorarachniophyte *Bigelowiella natans *[43]. Although phagotrophy has apparently been lost in their phototrophic offspring, the ancestor of euglenophytes was likely a eukaryovorous euglenid protist [26]. Morphological and ultra-structural analyses have demonstrated that *Peranema*-like eukaryovorous euglenids are similar in size to photosynthetic euglenophytes and are capable of ingesting algal prey by phagocytosis, which is presumably a necessary prerequisite for the eventual establishment of a secondary plastid [26]. A study using video microscopy illustrated two types of feeding in *P. trichophorum*: engulfment of a prey cell whole (phagocytosis) as well as sucking out the prey cell cytosol through the feeding apparatus (myzocytosis) [44]. The presence of 'red lineage' genes in *Peranema *(Additional file 2: Supplementary figs. S10, 11) supports the idea that the LGT might have occurred prior to the divergence of euglenophytes and at least some eukaryovorous euglenids, but after the branching of eukaryovorous euglenids from bacteriovorous ancestors [26]. In this scenario, the ancestor of euglenids might once have ingested algae with 'red lineage' secondary plastids as prey, and some genes might have been transferred from the prey to the nuclear genome of the ancestor of euglenids (Figure 2).

What kind of relationship might have existed between the above-mentioned LGT donors and recipients? Our data are consistent with the notion that these 'red lineage' genes were acquired multiple times independently via LGT from multiple sub-groups within the CR assemblage [43,45,46]. Under this view, some genes would have been lost while others happened to acquire a function in the host organism, perhaps ultimately contributing to the successful integration and functioning of the green algal endosymbiont that eventually became the current euglenophyte secondary plastid. Importantly, these genes were retained in the euglenophyte nuclear genome (at least in *E. gracilis*) without being replaced by the EGT-derived genes from the secondary green-algal endosymbiont, which gave rise to the 'modern-day' secondary plastids in extant euglenophytes.

A previous study illustrated a remarkable example of LGT from non-green algae to euglenophytes. Triose-phosphate isomerase (TIM) genes from euglenophytes have been shown to be monophyletic with red algal homologues, and only distantly related to green algae, Excavata and Chromalveolata, suggesting an LGT from red algae to euglenophytes [47]. Interestingly, we found that the TIM genes from the chlorarachniophyte *Bigelowiella natans*, a member of another algal lineage harboring secondary plastid of green algal origin, were also nested within the red algal clade (Additional file 2: Supplementary fig. S15A). A unique insertion found in red algae and euglenophytes [47] was also shared with the *B. natans *gene (Additional file 2: Supplementary fig. S15B). For consistency, we did not designate TIM as a 'red lineage' gene due to the absence of Chromalveolata genes in the euglenid plus red algae clade. Future analyses will hopefully uncover the true history of this enigmatic gene family.

### Endosymbiotic versus lateral gene transfer

Phagocytosis of prey cells, endosymbiosis and, ultimately, establishment of an organelle is a complex process that is difficult to understand from genomic data alone [48], and at the earliest stages of organellogenesis, the distinction between LGT and EGT is blurred. Nevertheless, several lines of evidence from our study are consistent with the possibility that at least some of the 'red lineage' genes in euglenids are derived from EGT rather than LGT. First, some of the 'red lineage' genes appear to have plastid-related functions (e.g., Additional file 2: Supplementary figs. S5, S7 and S8). Second, *Peranema *possesses 'red lineage' genes (Fig. 1B, Additional file 2: Supplementary figs. S10, S11) and the basic morphological characteristics believed to be necessary for establishment of a secondary plastid: *Peranema*-like euglenids are larger than other bacteriovorous and *Dinema*-like eukaryovorous euglenids, comparable in size and in the number of strips around the cell periphery (a well-established taxonomic character) to several phototrophic euglenophytes such as *Euglena *and *Eutreptiella *[26]. Genomic data from *Peranema*-like eukaryovores are currently very limited. Regardless, although morphological and phylogenetic analyses suggest that aplastidic (osmotrophic, bacteriovorous and eukaryovorous) euglenids are paraphyletic in euglenid phylogenies, *Peranema*-like eukaryovores are sister to phototrophic euglenophytes and possibly form a monophyletic group with them [49]. Third, although tree topologies are certainly dependent on taxon sampling (and the number of genome sequences available), the genes we have characterized can be interpreted as having come from an ancestral lineage within the broader CR assemblage, rather than one or a few specific lineages. On balance, it is thus possible that the incorporation (phagocytosis and perhaps endosymbiosis) of an alga harboring a secondarily derived 'red lineage' plastid could have occurred in a *Peranema*-like eukaryovorous ancestor (Figure 2).

Under the EGT scenario, retention of genes encoding plastid-targeted proteins such as PRK, ZEP and plastid-type FBP (Additional file 2: Supplementary figs. S5, S7 and S8, respectively) suggests that the secondary green plastid might have replaced the 'red lineage' tertiary plastid or succeeded it within a short period of time after plastid loss (Figure 2). This may be comparable to the situation seen in the 'green' plastid-harboring dinoflagellate *Lepidodinium chlorophorum*, where phylogenetic mosaicism of the nuclear-encoded plastid-targeted proteins derived from both the ancestral peridinin-type plastid, which is the most common plastid in dinoflagellates, and the 'new' secondary plastid of green algal origin, which is thought to have been acquired by plastid replacement [46].

From our data alone, it is difficult to distinguish unambiguously between the LGT and EGT hypotheses for any given gene. The data do, however, represent an interesting case study for discussing which hypothesis is most likely and under what conditions. If multiple phylogenetic trees suggest that the genes in question are most closely related to different organisms, the "independent LGT" scenario is most likely correct. However, if the tree topologies are consistent with one another and all the genes appear to be derived from the same source, then "ancient EGT" can be considered. Nevertheless, consistent tree topologies inferred from multiple genes/proteins do not necessarily prove that all the genes have a single origin, as phylogenetic artefacts can be misleading and taxon sampling is often insufficient to allow fine-scale resolution. On a gene-by-gene basis, LGT and EGT are indistinguishable on the basis of phylogenetic tree topology alone.

Endosymbiosis can be considered a specific and extreme case among numerous types of prey-predator relationships [50]. Assuming that genes are repeatedly transferred from the same prey organism and very close relatives during the transition from endosymbiont to organelle, an "ancient EGT" scenario entails stricter conditions on the nature of the organism engulfed by the host. Thus, based on parsimony, regardless of the number of transferred genes identified, "ancient EGT" would seem to be less likely than multiple LGTs form a single donor lineage, or "single-origin LGT". However, this parsimony-based argument does not necessarily apply in every biological context. For instance, when the host cell retains an endosymbiont that shares a recent common ancestor with the source organism of the transferred genes, "EGT" would be regarded as the most parsimonious interpretation. Even when the host cell does not possess such an endosymbiont, if the frequency of the gene transfer correlates with the length of the period when the prey is retained in the host cell, increasingly large numbers of transferred genes would increasingly favor the "EGT" scenario. One can also argue that if the presumed functions of the transferred genes are assumed to be reflective of the relationship between the source organisms and the host cell, and if those genes possess plastid-related functions, "ancient EGT" would also seem to be a likely scenario. There are at present no known criteria with which to quantify and compare the probabilities of LGT and EGT from genomic data, and further study will be necessary to verify whether the above assumptions are biologically reasonable.

### Alternative explanations and limitations in data interpretation

As noted above, combined with the potential to be misled by phylogenetic artifact, insufficient taxon sampling is also a concern when interpreting the phylogenies presented herein. A previous study suggested that the plastid-bearing ancestor of *E. gracilis *is related to the prasinophyte genus *Pyramimonas *(Pyramimonadales) [9], from which a complete nuclear genome sequence is not yet available. If so, the *E. gracilis *genes would be expected to be basally branching within the green plant clade, or branching between green plants and other primary plastid-containing lineages (red algae and glaucophytes). However, only two genera, *Ostreococcus *and *Micromonas *(Mamiellales), were included in our database among prasinophytes, making interpretation difficult.

A recent genome-wide phylogenetic study demonstrated the presence of over 100 genes of apparent algal affinity, probably derived from LGT events, in the choanoflagellate *Monosiga brevicollis *[51]. The red algal-like glucokinase analyzed herein (Additional file 2: Supplementary fig. S3), which was not identified in the previous study [51], may provide another example of LGT-derived algal genes in *M. brevicollis*. Nevertheless, given that other eukaryotes (animals, fungi, land plants and some excavates) possess a different type of enzyme for phosphorylating hexose [35,36], our data do not rule out the possibility of differential loss of multiple gene families with overlapping functions early in eukaryotic evolution.

It is also important to recognize that the *Euglena *and *Peranema *EST data are far from complete gene repertoires and that nuclear genome sequences, especially of basally branching green algae and euglenids, would be helpful to better resolve the early history of plastids in euglenophytes and other photosynthetic eukaryotes. Wider and richer taxon sampling will also help to reduce the impact of phylogenetic artifacts, e.g., long-branch attraction, stochastic variation or directional biases of evolutionary signals.

As discussed above, recent studies have suggested that the ancestor of Chromalveolata possessed a considerable number of genes showing affinity to green plants [2,5,25,32]. If chromalveolates are a monophyletic group, how would such 'green'-type genes be expected to behave in phylogenetic trees relative to the EGT-/green algal-derived genes of euglenids and chlorarachniophytes? 'Green'-type genes in euglenids and CR could be monophyletic due to phylogenetic artifacts (as could the 'red'-type ones) and we set aside trees in which their monophyly was weakly supported and/or the phylogenetic patterns were too ambiguous. It is notable that phylogenetic patterns such as those seen in the FBT and ZEP trees (Additional file 2: Supplementary figs. S6 and S7, respectively) could be interpreted as a result of LGT from CR specifically to prasinophytes, which is in the opposite direction to the model proposed in previous studies [25,32]. Thus, it is important to recognize that the directionality of LGT events can be difficult to discern with confidence and greatly impact how we interpret global patterns of plastid gain and loss. Regardless, despite numerous uncertainties our data clearly indicate that euglenid nuclear genomes are evolutionary mosaics, the result of a complex past in which LGTs from (i) CR to euglenids, (ii) from green plants to CR (and the reverse), as well as (iii) EGTs from red algae to CR and (iv) from green algae to euglenids, appear to be overlaid upon the 'host lineage' phylogeny. It is essential that our understanding of the evolutionary histories of these be reevaluated regularly and cautiously as more genomic data accumulate.

## Conclusions

We have identified a number of 'red lineage' genes in the phototrophic euglenophyte *E. gracilis*, an organism that harbors a green algal-derived secondary plastid, as well as in the plastid-lacking eukaryovorous euglenid *P. trichophorum*. It is likely that these genes have been acquired via eukaryote-to-eukaryote LGT, giving rise to a complex pattern of genome mosaicism in euglenids. The possible sources of these genes are from prey organisms, and, possibly, the presence of a cryptic 'red lineage' tertiary endosymbiont in an ancestral euglenid. Such LGT- and/or EGT-derived genes may have contributed to the successful integration and functioning of the green algal secondary plastid in modern-day euglenids.

## Methods

### cDNA library and sequencing

*P. trichophorum *cells were co-cultured with *Chlorogonium *sp. as described previously [52]. The total RNA was extracted using SV Total RNA Isolation (Promega, WI, USA), and a cDNA library for *P. trichophorum *was constructed and end-sequenced (TAKARA BIO Inc., Shiga, Japan). The prey *Chlorogonium *cells were depleted in the cultures when the *Peranema *cells were collected. For *E. gracilis *genes, 3' end sequences were amplified by rapid amplification of cDNA ends (RACE) using the Omniscript RT kit (Qiagen, CA, USA). The *E. gracilis *LepA and *P. trichophorum *ADP/ATP transporter gene fragments were deposited in DDBJ/EMBL/GenBank under the accession numbers AB617525 and AB617526, respectively.

### Data mining and phylogenetic analysis

The genome sequence data and phylogenetic tools used in the similarity search and tree construction were as described in our previous study [28]. The EST sequences of *E. gracilis *were obtained from TBestDB (http://tbestdb.bcm.umontreal.ca/) and all other sequences were from the NCBI GenBank refseq database (http://www.ncbi.nlm.nih.gov/), the JGI genome database (http://genome.jgi-psf.org/) and the *Galdieria sulphuraria *whole genome data (A.P.M. Weber, unpublished). We excluded amitochondrial and/or parasitic eukaryotes, which might cause long branch attraction artifacts due to unusual nucleotide compositions and accelerated rates of sequence evolution [53,54].

For the first screening step, amino acid query sequences derived from *E. gracilis *genes (8651 queries) were automatically subjected to BLAST searches against the GenBank non-redundant (nr) database using NCBI netblast and EFetch utilities, extracting the genes showing the E-value smaller than 10e-5 to 'Viridiplantae' by BLASTP. For the second step, the selected query sequences (2632 queries) were subjected to BLASTP analysis against 'refseq-protein' to fetch homologous sequences with E-values less than 0.001, up to 500 hits at a maximum. Multiple alignments, phylogenetic tree constructions and laterally transferred gene mining were carried out using a gene mining pipeline that we developed in a previous study [28]. Briefly, multiple alignments were then performed using MUSCLE [55], followed by automated removal of indel-rich sites and taxa. Bootstrapped neighbor-joining trees were produced using QuickTree [56]. To diminish the sampling bias, all the OTUs except for one representative OTU in a monophyletic clade exclusively composed of OTUs from a single genus were removed, and the trees were re-constructed for calculating the distance between the query and any taxon of interest on the tree. In addition to the automatic process, trees for genes previously published as the putative photosynthetic endosymbiont-derived genes, but not detected in our analysis, were manually re-constructed.

Candidate 'red lineage' genes in *E. gracilis *were manually selected, and their homologues were collected based on the BLASTP scores, and then subjected to multiple protein sequence alignments using MUSCLE. Phylogenetic analyses were performed with maximum likelihood (ML) using RAxML [57] and with Bayesian interference (BI) using MrBayes [58]. ML and BI were based on the WAG substitution matrix, which gave high scores for all proteins in model selection using ModelGenerator [59], with options of four gamma-distributed rate categories and estimate of invariable sites (plus empirical base frequencies in ML). ML branch support was evaluated with 1000 bootstrap replicates, and BI posterior probability values were calculated from the MCMC run data, which summarized when the average standard deviation of split frequencies reached less than 0.01. Except for the trees of which monophyly was confirmed by previous studies, threshold values to assess the monophyly of *E. gracilis *gene clades were 70% on ML bootstrap or 0.9 on BI posterior probability values.

## Authors' contributions

SM designed the study, conducted the analysis and wrote the manuscript. TS carried out the *Peranema *culturing. APMW contributed the phylogenomic analysis. HN helped designing and conducting the study. JMA and HN contributed to the data interpretation and manuscript preparation. All authors read and approved the final manuscript.

## Supplementary Material

Additional file 1 **Taxonomical distribution of the 'closest gene' to the *E. gracilis *genes in distance on the first screening NJ trees**. Click here for file

Additional file 2 **The HPT protein family**. A, RAxML tree of the HPT proteins. The RAxML bootstrap values (upper) and the MrBayes posterior probability values (lower) are shown on each branch. Thick branches represent BI and ML values not lower than 100 and 95, respectively. Different phylogenetic affiliations are represented as follows: blue, Excavata; orange, Chromalveolata plus Rhizaria; magenta, red algae; green, green plants; gray, unikonts; sky blue, cyanobacteria. B, Partial amino acid alignments showing the unique Indels in the HPT family proteins. RAxML tree of hypothetical proteins. RAxML tree of GLK proteins. ClpP protein family. A, RAxML tree of ClpP proteins. B, Partial amino acid alignments showing the conserved amino acid residues in the ClpP family proteins. RAxML tree of PRK proteins. RAxML tree of FBT proteins. RAxML tree of ZEP proteins. RAxML tree of paltidic FBP proteins. RAxML tree of cytosolic FBP proteins. RAxML tree of GND proteins. Glaucophytes are represented in blue-green. RAxML tree of ADP/ATP transporter proteins. RAxML tree of LepA proteins. RAxML tree of MAT proteins. Fatty acid desaturase protein family. A, RAxML tree of fatty acid desaturase proteins. B, Partial amino acid alignments showing the unique Indels in the fatty acid desaturase family proteins. TIM protein family. A, RAxML tree of TIM proteins. B, Partial amino acid alignments showing the unique Indels in the TIM family proteins. Click here for file
