# Supplementary material for: Eukaryote-to-eukaryote gene transfer gives rise to genome mosaicism in euglenids
Source: BMC Evol Biol. 2011 Apr 18;11:105. doi: 10.1186/1471-2148-11-105 (PMC3101172; doi:10.1186/1471-2148-11-105)
Supplement: Additional file 1 — Taxonomical distribution of the 'closest gene' to the E. gracilis genes in distance on the first screening NJ trees. [file 1471-2148-11-105-S1.PDF]

Supplementary table 1.
Taxonomic distribution of the 'closest gene' to the *E. gracilis* genes in distance on the first screening NJ trees

Using the 2602 NJ trees in which at least one Viridiplantae genes were included, the OTU connected to the *E. gracilis* homologue with the shortest distance was calculated. *B. natans* (Rhizaria) genes were not included at this stage. Note that the *E. gracilis* gene and the 'closest' are not always in a monophyletic clade on each tree.

Higher rank	Taxon	number of genes	
< Excavata >			
	Euglenozoa	410	
	Heterolobosea	107	
	Diplomonadida	8	
	Parabasalidea	24	
< Archaeplastida >			
	Viridiplantae	528	
	Glaucocystophyceae	1	
	Rhodophyta	69	
< CR group >			
	stramenopiles	288	’red lineage’
	Alveolata	84	total 621
	Cryptophyta	15	
	Haptophyceae	165	
	Rhizaria	1	
< unikonts >			
	Amoebozoa	62	
	Choanoflagellida	62	
	Fungi	129	
	Metazoa	274	
< eubacteria >			
	Cyanobacteria	42	
	Acidobacteria	5	
	Actinobacteria	34	
	Bacteroidetes	22	
	candidate division TG1	1	
	Chlamydiae	5	
	Chlorobi	3	
	Chloroflexi	6	
	Deinococcus-Thermus	8	
	Firmicutes	25	
	Lentisphaerae	3	
	Planctomycetes	8	
	Proteobacteria	169	
	Spirochaetes	7	
	Tenericutes	2	
	Verrucomicrobia	26	
< others >			
	dsDNA viruses, no RNA stage	3	
	Crenarchaeota	3	
	Euryarchaeota	2	
	Korarchaeota	1	
	total	2602	
