# Supplementary material for: Eukaryote-to-eukaryote gene transfer gives rise to genome mosaicism in euglenids
Source: BMC Evol Biol. 2011 Apr 18;11:105. doi: 10.1186/1471-2148-11-105 (PMC3101172; doi:10.1186/1471-2148-11-105)
Supplement: Additional file 2 — The HPT protein family. A, RAxML tree of the HPT proteins. The RAxML bootstrap values (upper) and the MrBayes posterior probability values (lower) are shown on each branch. Thick branches represent BI and ML values not lower than 100 and 95, respectively. Different phylogenetic affiliations are represented as follows: blue, Excavata; orange, Chromalveolata plus Rhizaria; magenta, red algae; green, green plants; gray, unikonts; sky blue, cyanobacteria. B, Partial amino acid alignments showing the unique Indels in the HPT family proteins. RAxML tree of hypothetical proteins. RAxML tree of GLK proteins. ClpP protein family. A, RAxML tree of ClpP proteins. B, Partial amino acid alignments showing the conserved amino acid residues in the ClpP family proteins. RAxML tree of PRK proteins. RAxML tree of FBT proteins. RAxML tree of ZEP proteins. RAxML tree of paltidic FBP proteins. RAxML tree of cytosolic FBP proteins. RAxML tree of GND proteins. Glaucophytes are represented in blue-green. RAxML tree of ADP/ATP transporter proteins. RAxML tree of LepA proteins. RAxML tree of MAT proteins. Fatty acid desaturase protein family. A, RAxML tree of fatty acid desaturase proteins. B, Partial amino acid alignments showing the unique Indels in the fatty acid desaturase family proteins. TIM protein family. A, RAxML tree of TIM proteins. B, Partial amino acid alignments showing the unique Indels in the TIM family proteins. [file 1471-2148-11-105-S2.PDF]

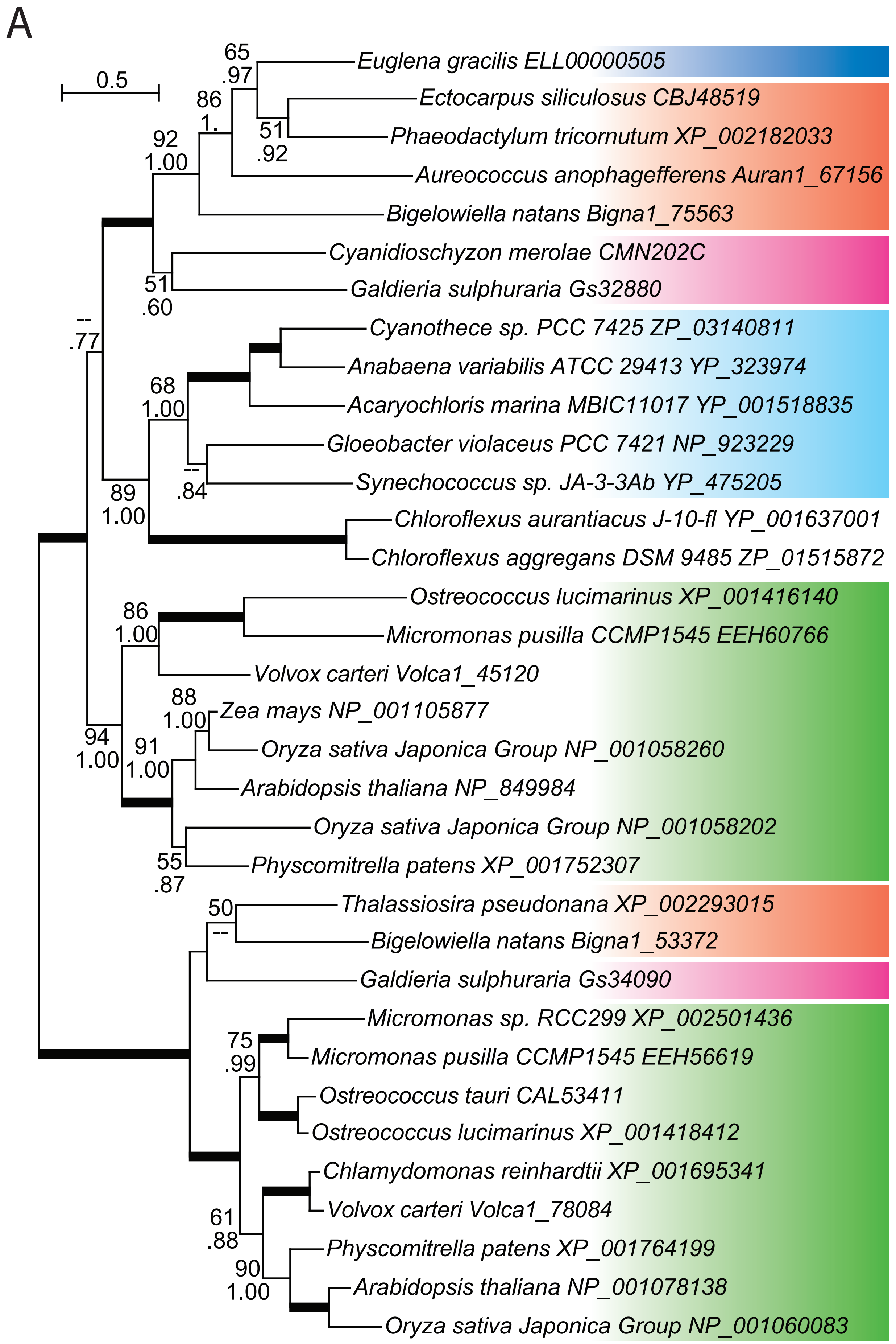

Sup. Fig. 1 Homogentisate phytyltransferase

B

Group	Organism	154				194
	Euglena	LAAGLALVPL	VYAQVAALQP	FATPALQATL	VGSALLGTVY	S--LPPFRLK
CR+Red	Ectocarpus	LLAGAVL---	----GLAPCS	LGSPGLALTV	ILSVLIGTVY	S--LPPFRLK
CR+Red	Aureococcus	LAGAALL---	----GCDAR-	LGSEPLRRVL	LGSALLGFAY	S--APPLRLK
CR+Red	Phaeodactylum	LFGSLWL---	----GAANPV	FSTQGLNVAL	WGSGILGTMY	S--LPPFRLK
CR+Red	Bigelowiella	LIAGLLI---	----GFLPG-	VTTAPLQFTL	VASGLLGTLY	S--LPPFRFK
CR+Red	Cyanidioschyzon	LLGSFSL---	----GFWLP-	QSTAALRFAL	VASCILGTLY	S--LPPIRLK
CR+Red	Galdieria	GSLGLIL---	----GLVLP-	KTSVPLIGTL	FGSTLLGSMY	S--IPPIRLK
Green plants	Ostreococcus	CSSSVIC---	----GTL---	TQSFHLLTTL	VLSLLLGVIY	STDFKLLRWK
Green plants	Micromonas	TTSSLVL---	----GMM---	SGSSALLWAL	VLSLILGIVY	SVDYPGLRWK
Green plants	Volvox	AGVSVWI---	----GVA---	SGSCALLTTL	LVSLVLGVLY	SVELPFMRWW
Green plants	Oryza	LIMSIAI---	----GIR---	SKSAPLLCAL	FISFFLGSAY	SVDAPLLRWK
Green plants	Zea	AAMSFGL---	----GWA---	VGSQPLFWAL	FISFVLGTAY	SINLPYLRWK
Green plants	Arabidopsis	SIMSFWL---	----GWI---	VGSWPLFWAL	FVSFMLGTAY	SINLPLLRWK
Green plants	Physcomitrella	AAVSVGM---	----GFY---	VESPPLLWAL	LVSLVLGTAY	SADLPFLRWK
Eubacteria	Acaryochloris	GLLALGL---	----SA----	IQGYRLLWTV	GLSMLMGTVY	S--IPPIRLK
Eubacteria	Cyanothece	GVVALLI---	----AA----	WEGPFLLATV	GSSLIIGTAY	S--LPPVRLK
Eubacteria	Anabaena	GILALAL---	----AW----	LNGPYLFGMV	AVSLAIGTAY	S--LPPIRLK
Eubacteria	Gloeobacter	GVAALVI---	----A-----	LQNLYLFATV	AASVLIGTAY	S--LPPLRLK
Eubacteria	Synechococcus	GVASILL---	----AL----	TGIPYLLLTV	LLSNGIGTAY	S--LPPLRLK
Eubacteria	Chloroflexus	LVIALT----	----GAA---	VLSTALLITV	SVIALIGSLY	S--LPPLRLK

Supplementary Fig. 1
Homogentisate phytyltransferase (continued)

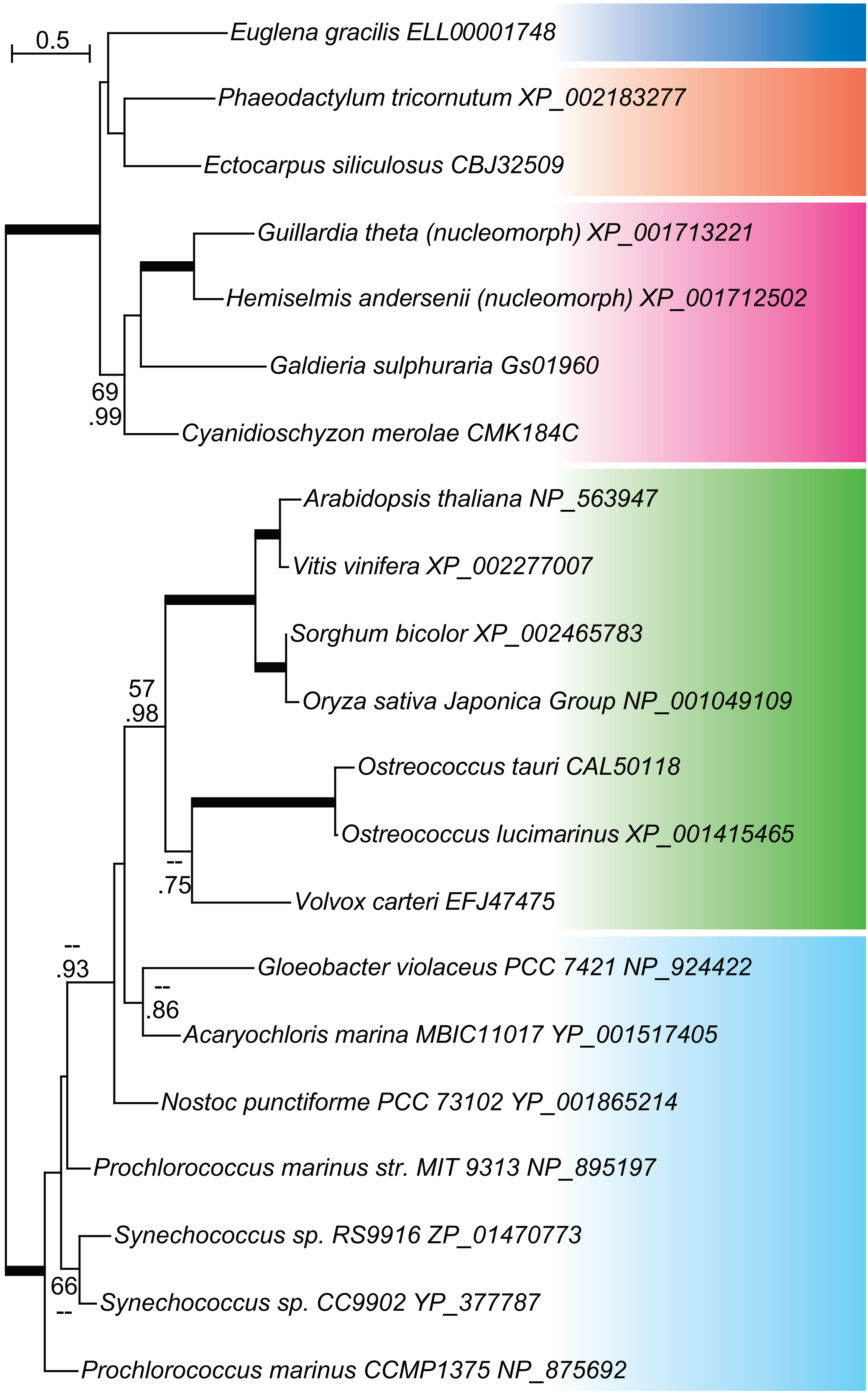

Sup. Fig. 2 Hypothetical protein

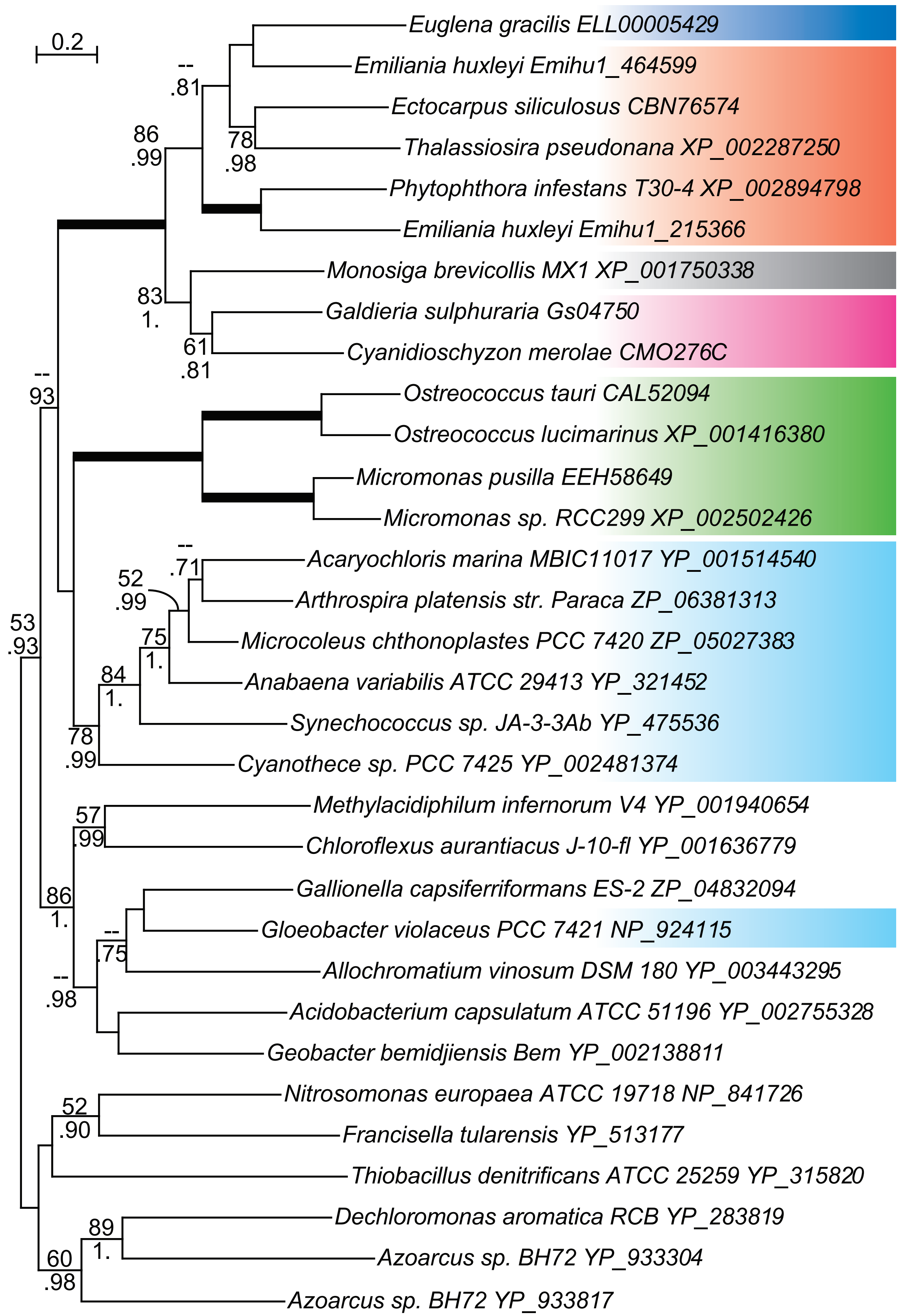

Sup. Fig. 3 Glucokinase

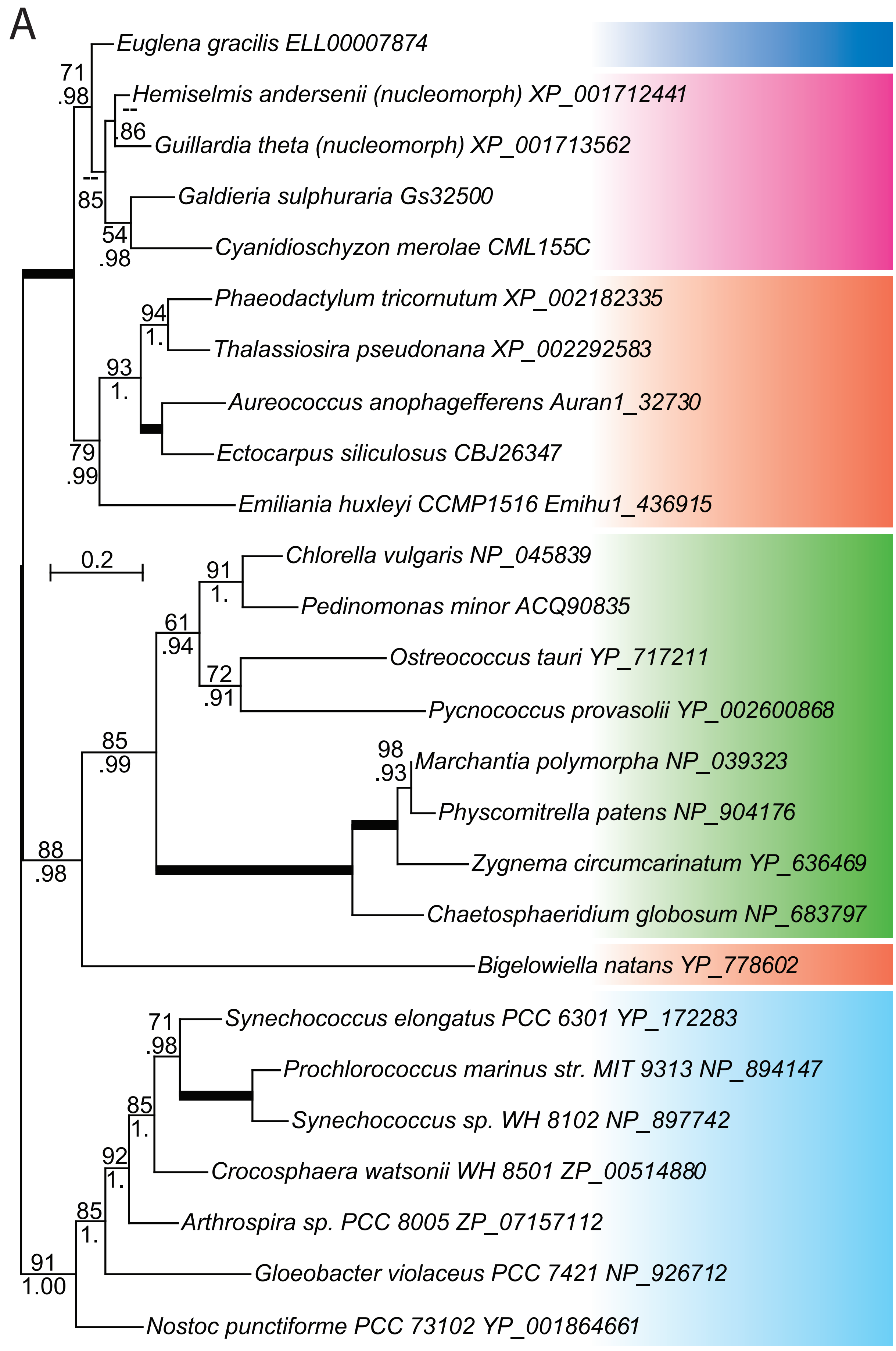

Sup. Fig. 4 Clp peptidase, proteolytic subunit

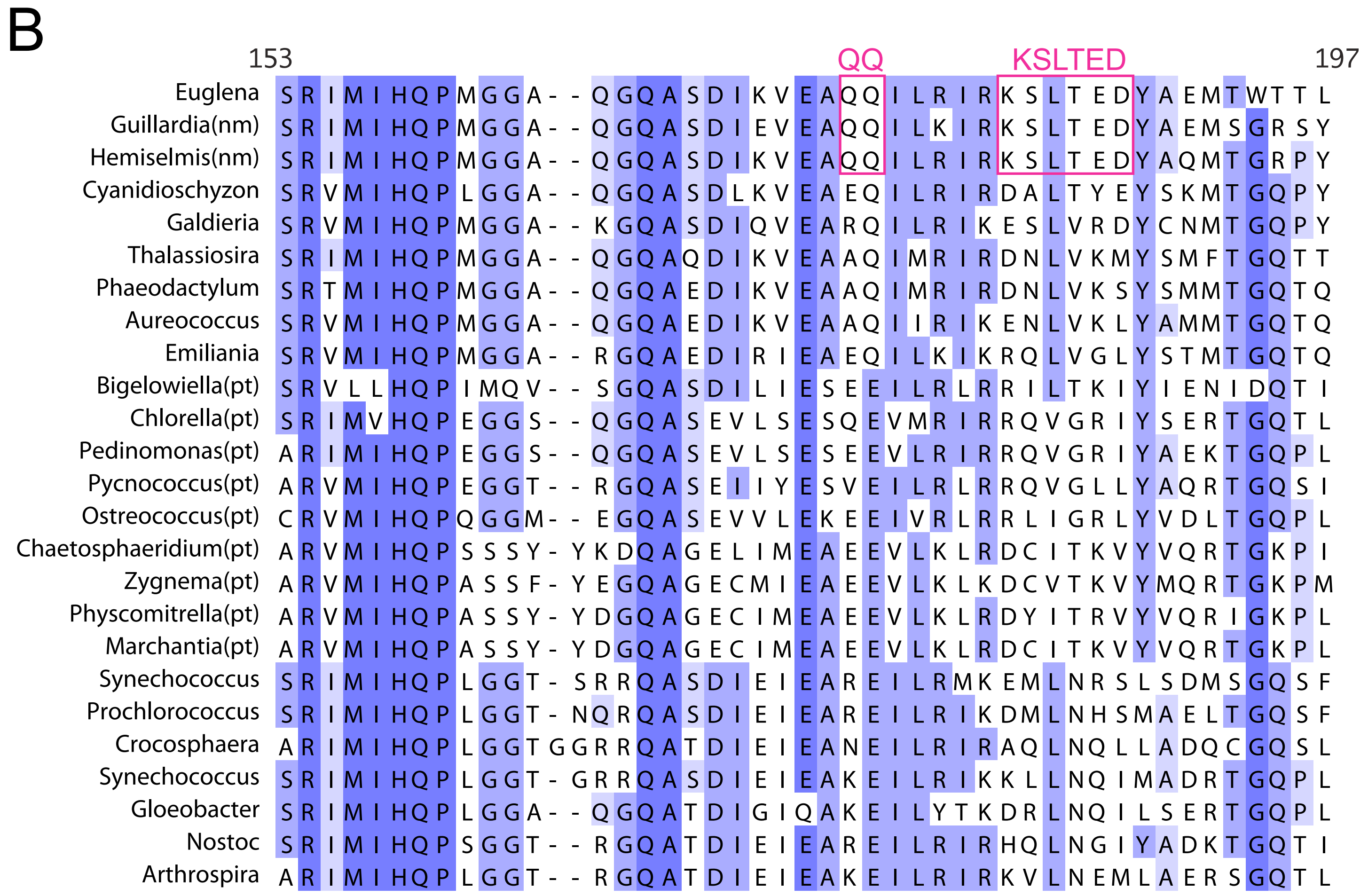

Supplementary Fig. 4

Clp peptidase, proteolytic subunit (continued)

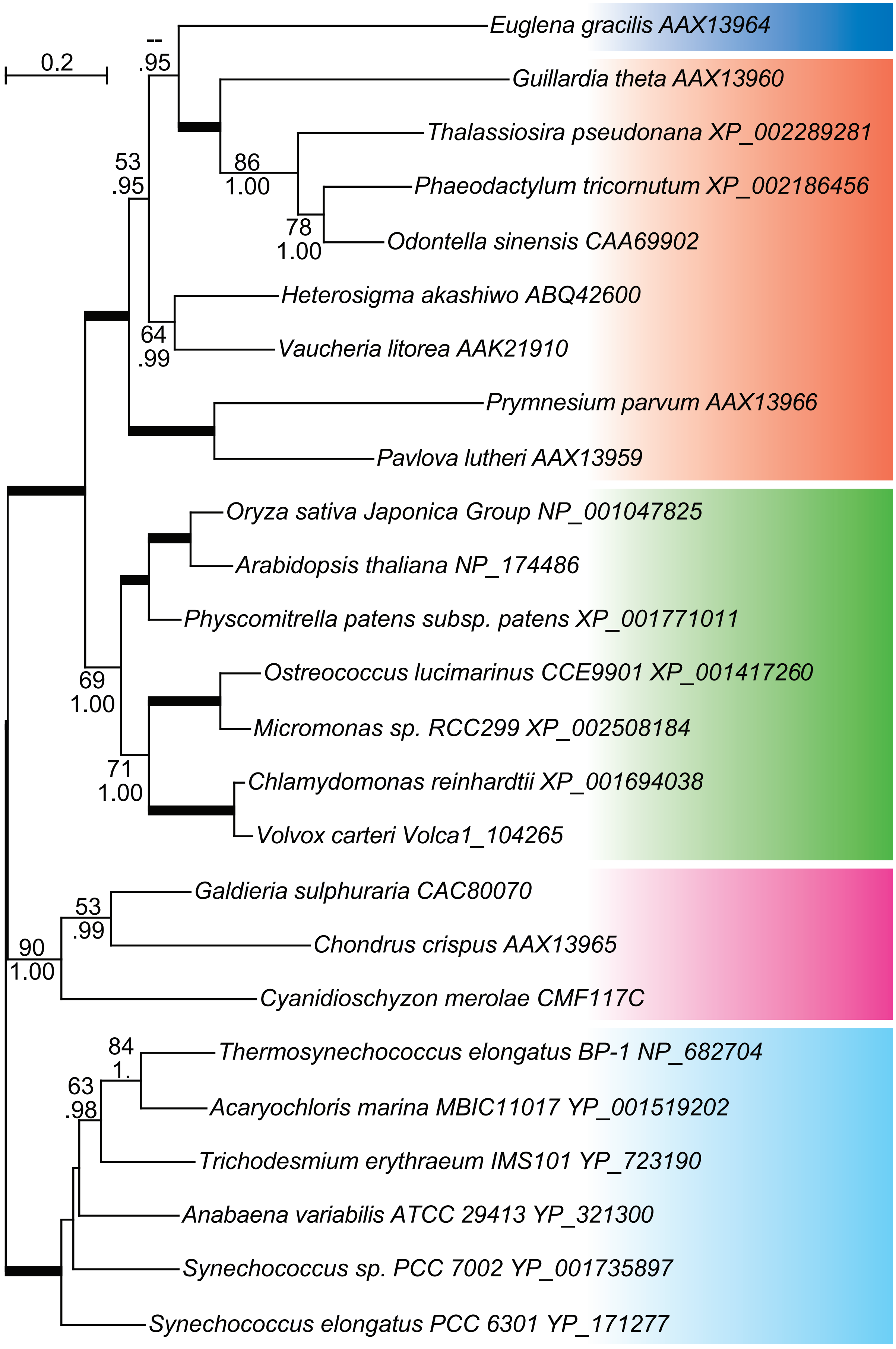

Sup. Fig. 5 Phosphoribulokinase

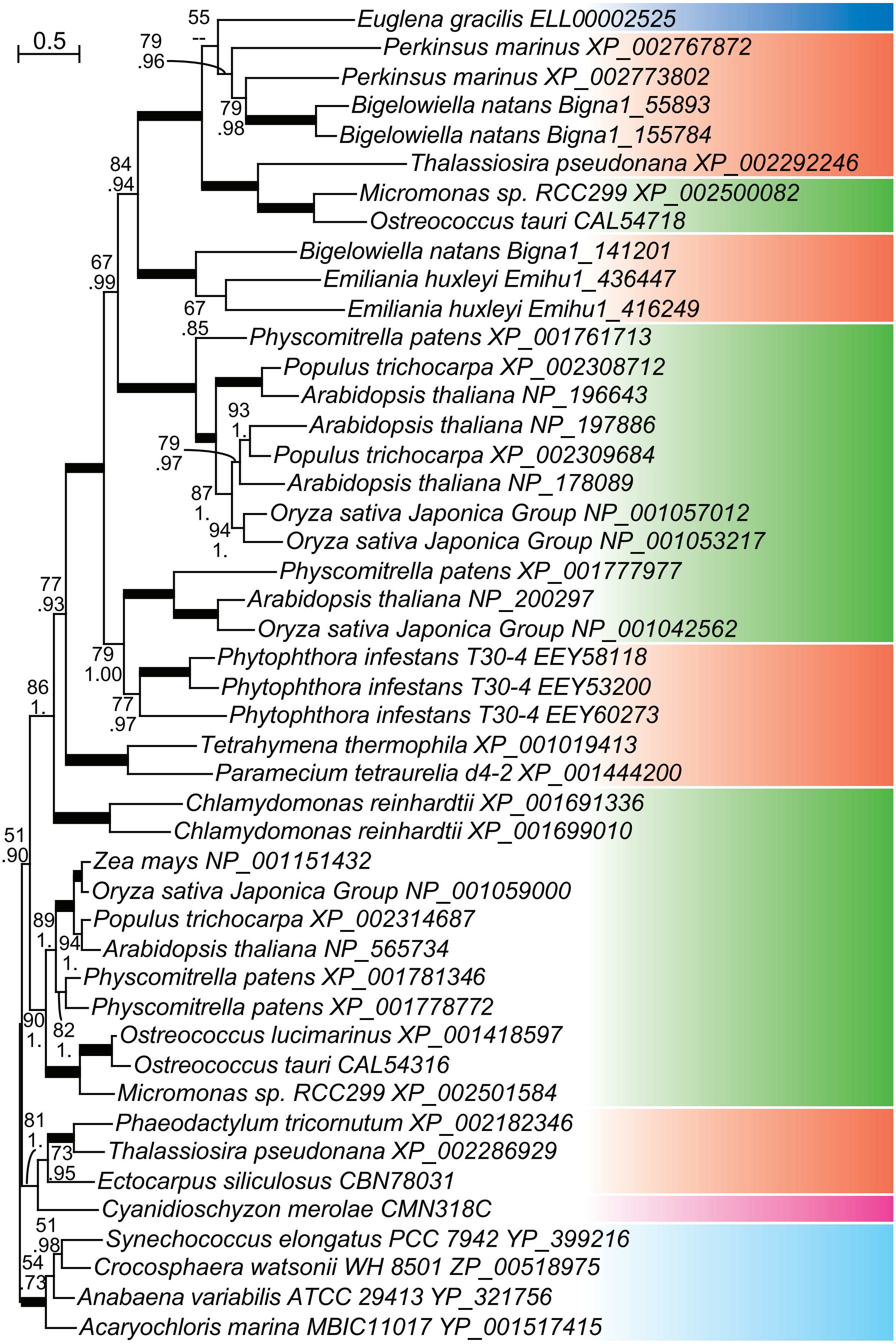

Sup. Fig. 6 Folate-biopterin transporter

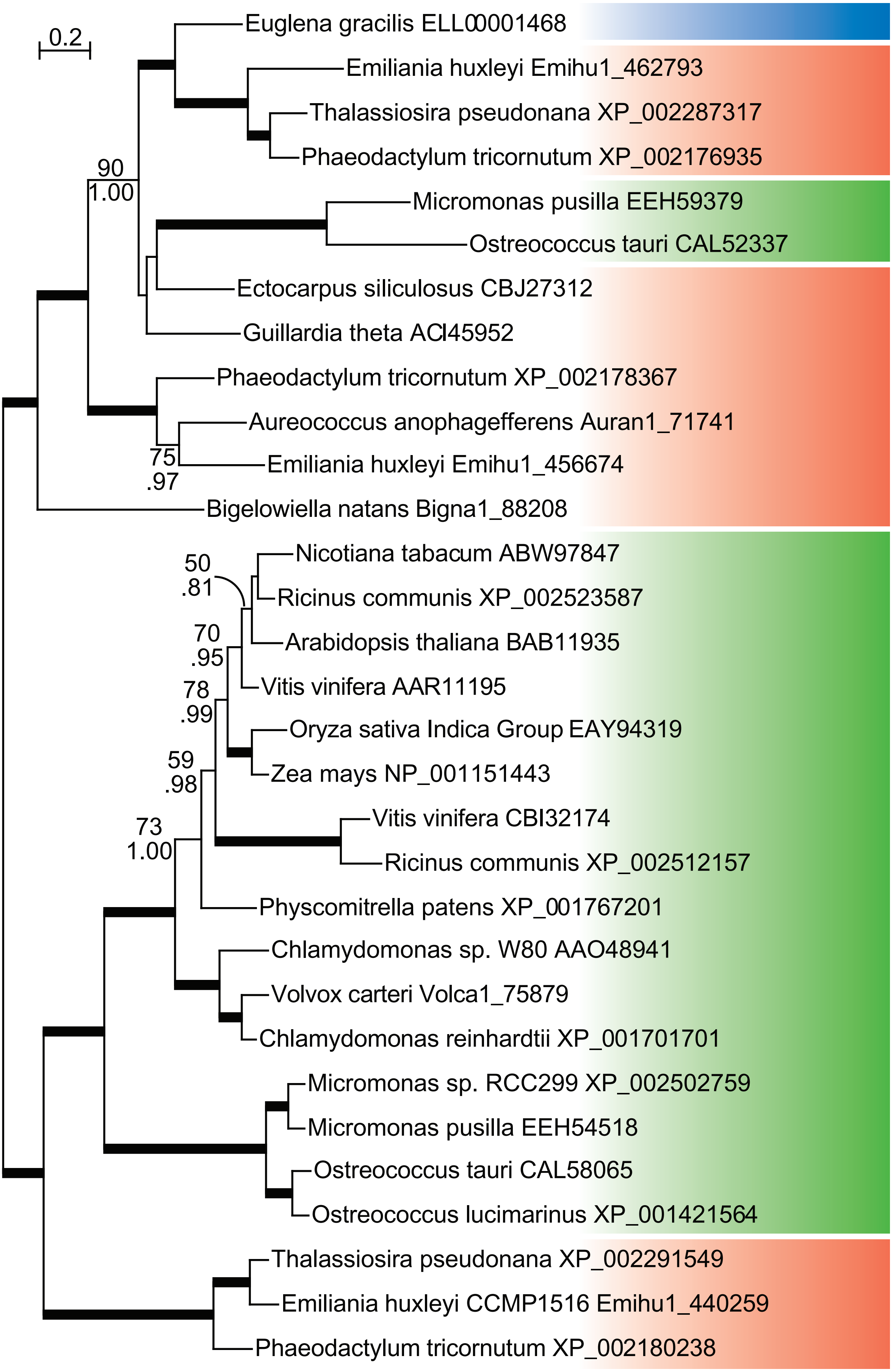

Sup. Fig. 7 Zeaxanthin epoxidase

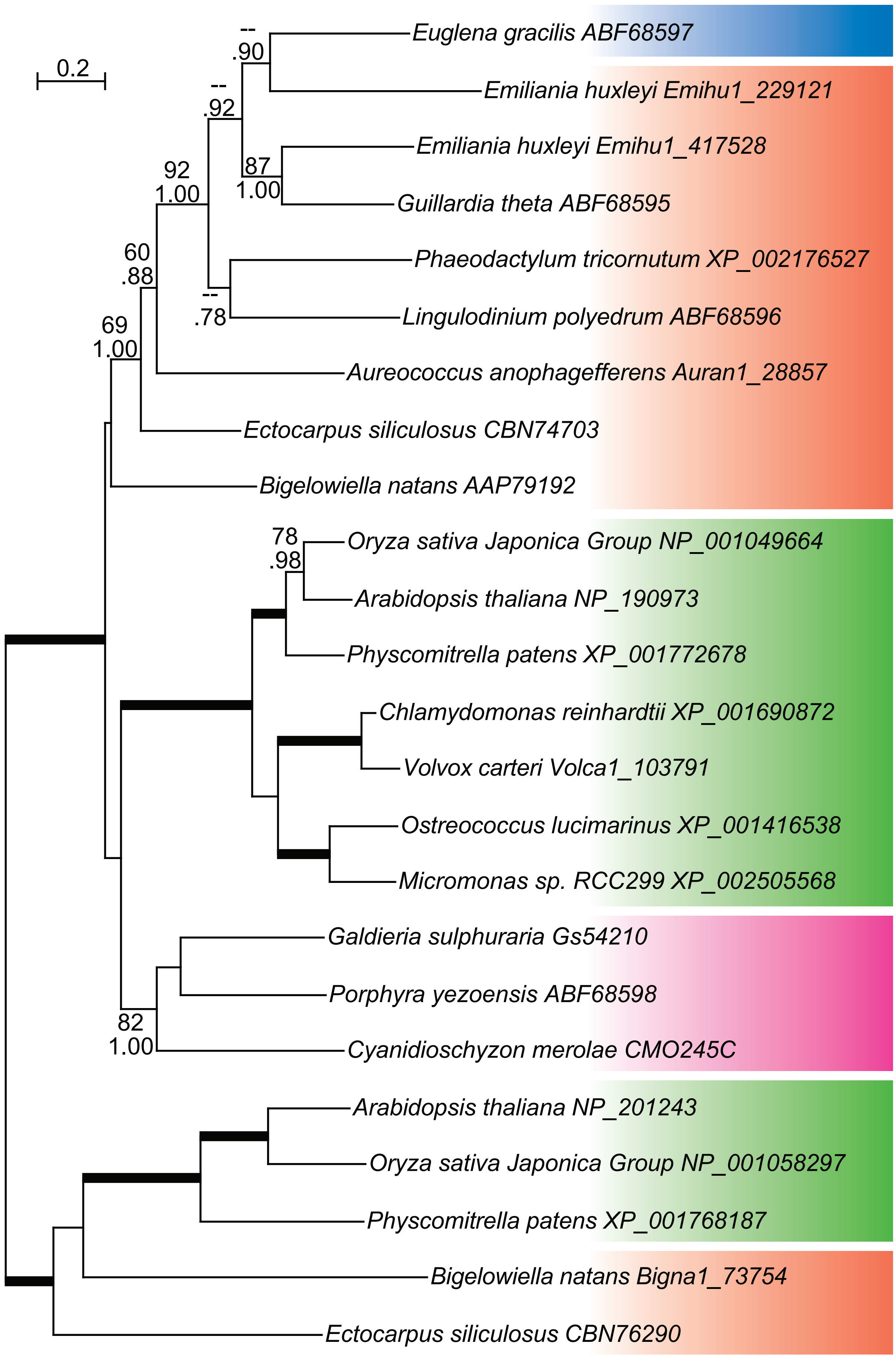

Sup. Fig. 8 FBPase, plastidic

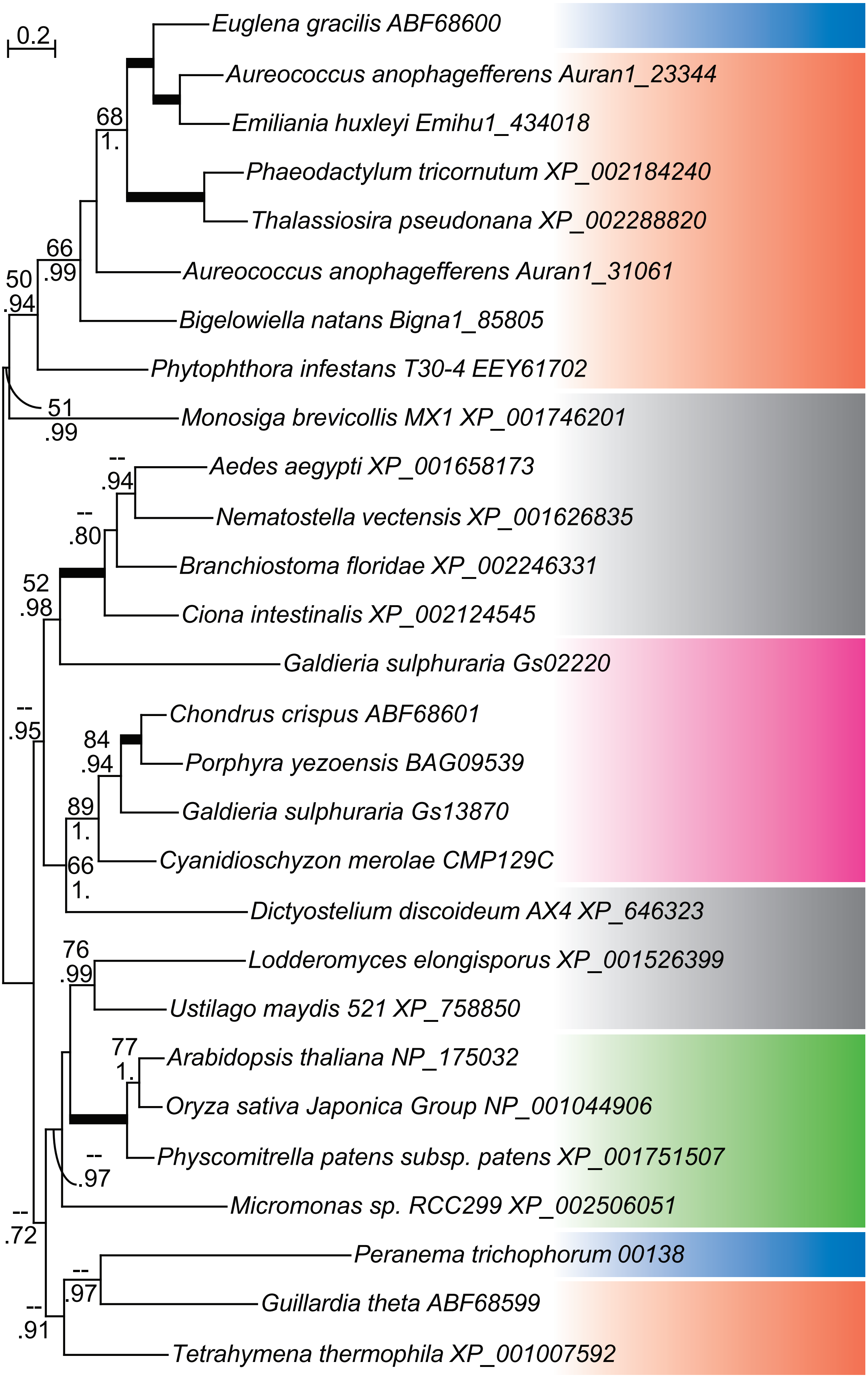

Sup. Fig. 9 FBPase, cytosolic

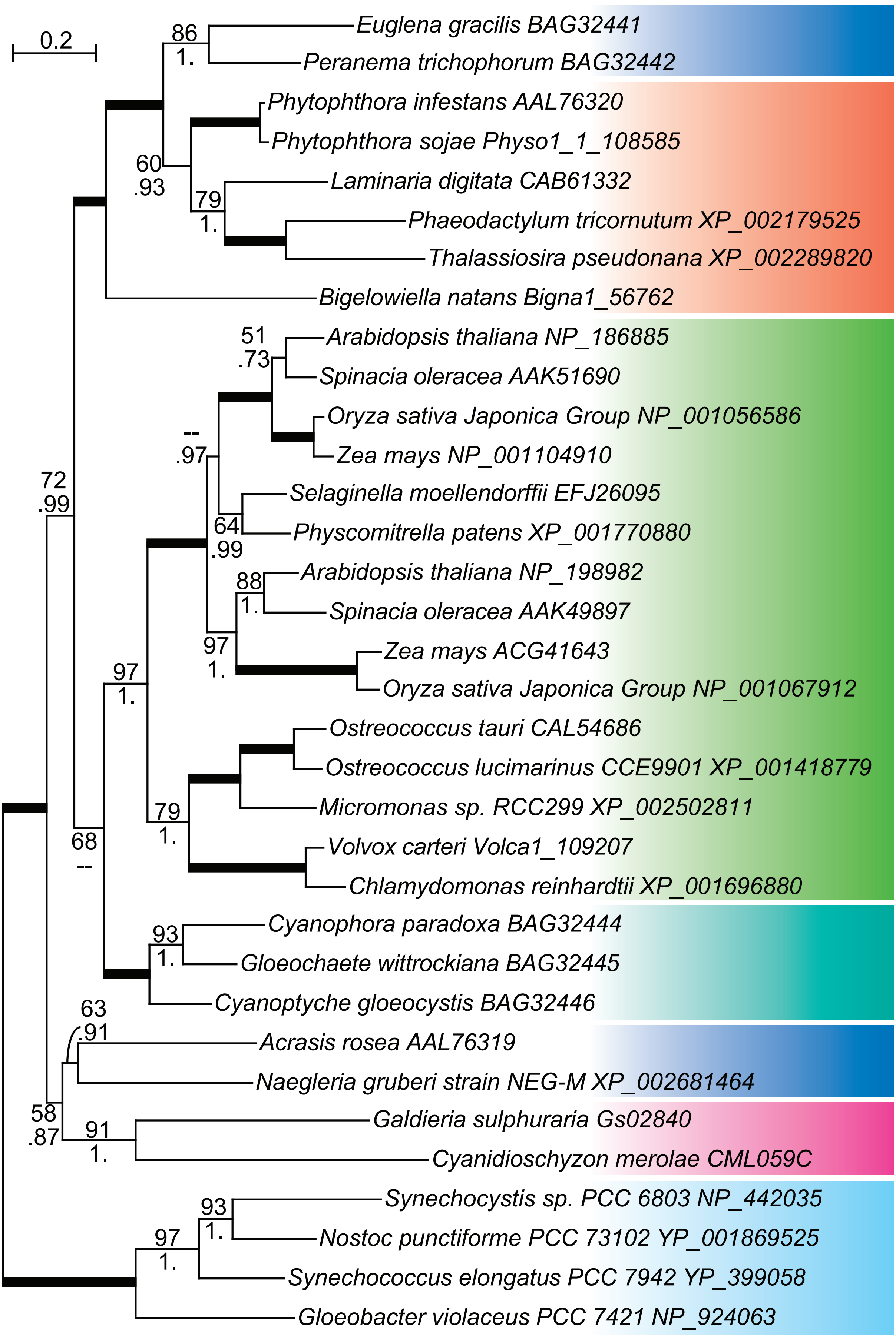

Sup. Fig. 10, 6-phosphogluconate dehydrogenase

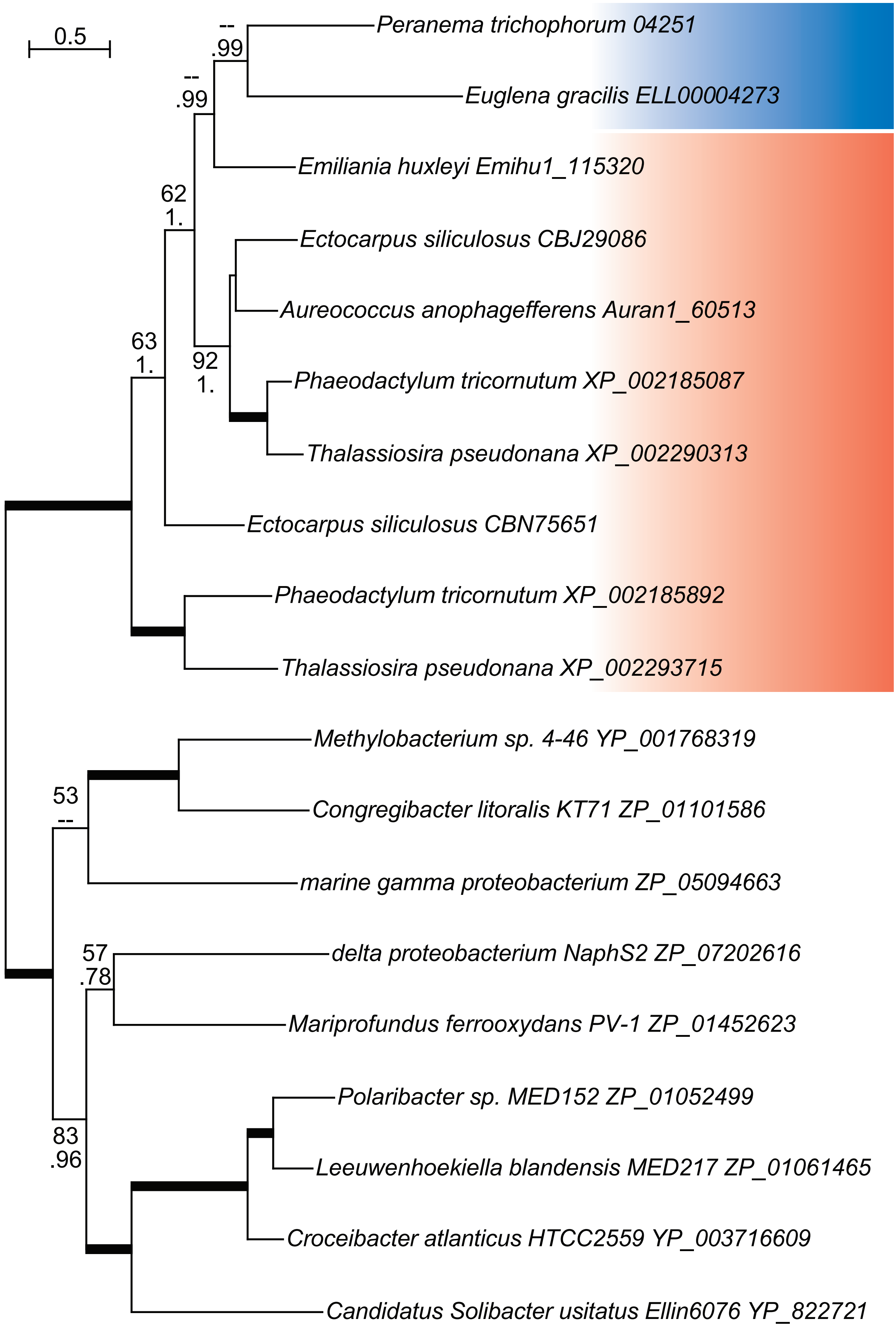

Sup. Fig. 11 Nucleotide transporter

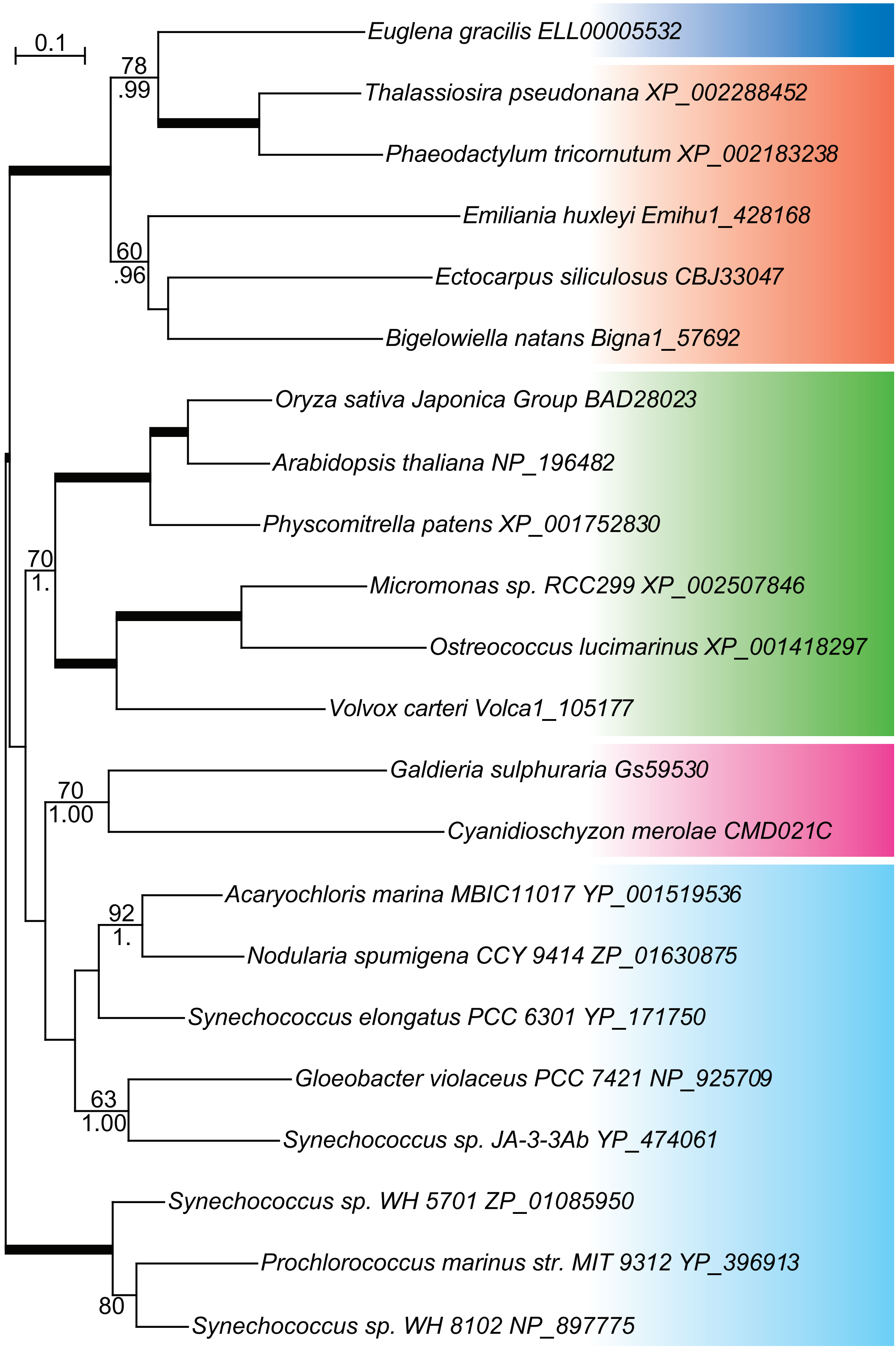

Sup. Fig. 12 GTP-binding protein LepA

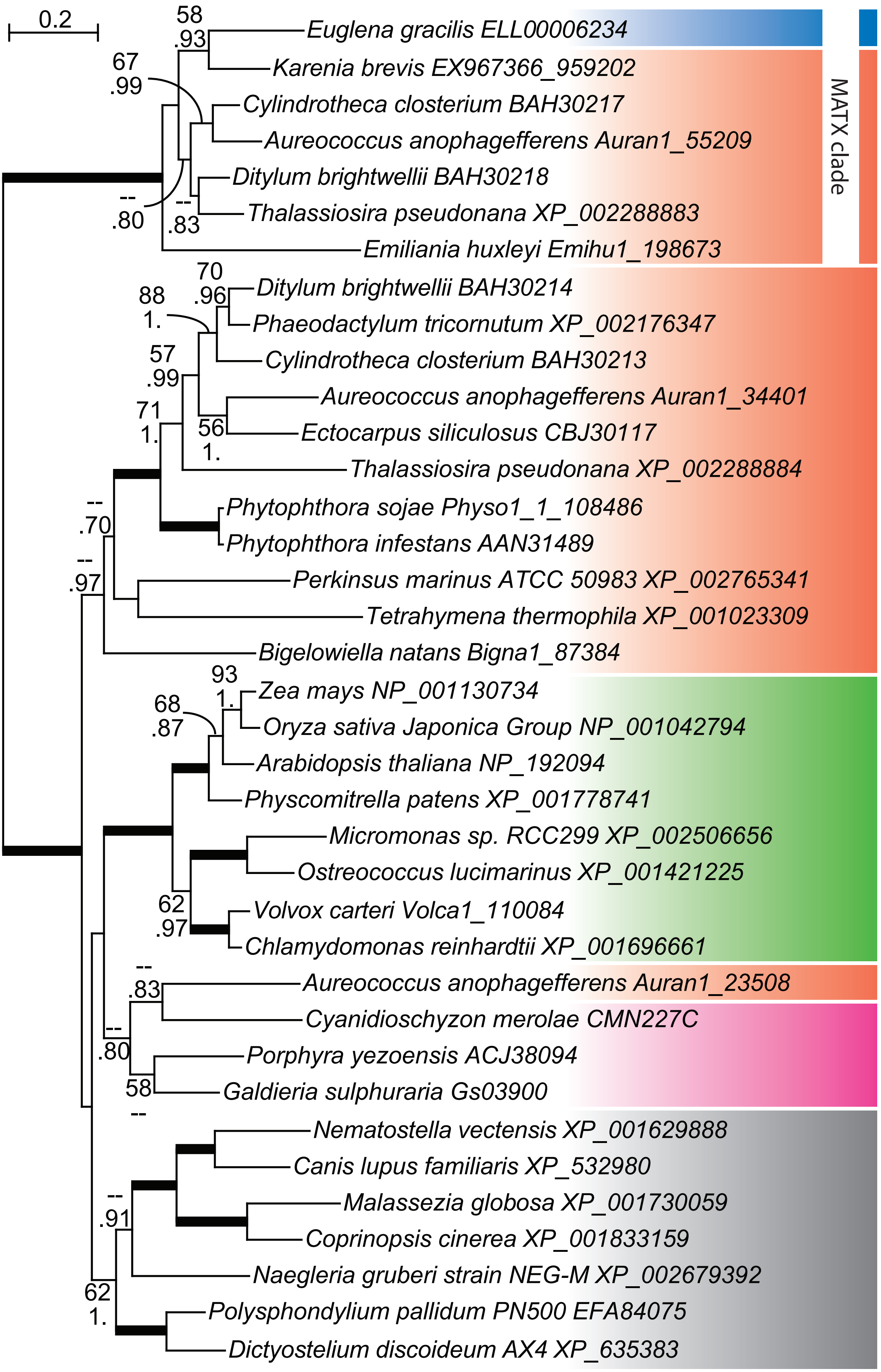

Sup. Fig. 13 Methionine adenosyltransferase

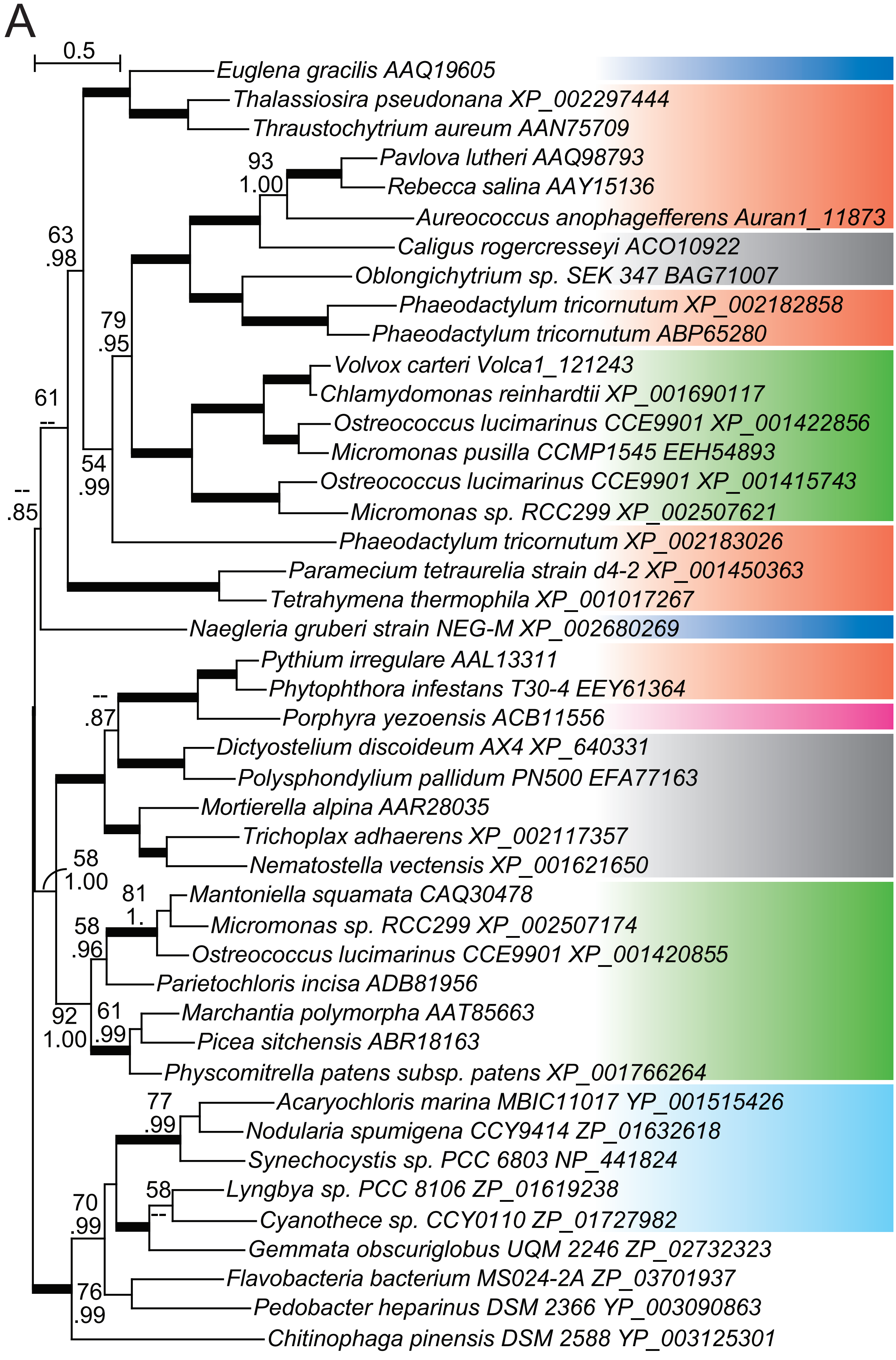

Sup. Fig. 14 Fatty acid desaturase

B

```
                                   239                                               288
          Euglena                  EYQHVIGHHQ YTNLVS---- ---------D TLFSLPENDP DVFSSY-PLM R
CR        Thalassiosira            ELQHMLGHHP YTNVLDGVEE ERKERGEDVA LEEKDQESDP DVFSSF-PLM R
CR        Thraustochytrium         EMQHVLGHHP YTNLIEMENG LAKVKGADVD PKKVDQESDP DVFSTY-PML R
Excavata  Naegleria                KMIHSVGHHV NTN------- ---------- ----IEERDP DIHTGE-PHF R
Excavata  Trypanosoma              IIHHDFIHHI YTN------- ---------- ----DPHRDL DLE--I-PLL R
Excavata  Trypanosoma              IIHHDFIHHV YTN------- ---------- ----EPHHDL DL--DI-PLL R
Excavata  Leishmania               IVNHDYVHHV YTN------- ---------- ----EPGRDA DLE--I-PLL R
CR        Pavlova                  LQEHVVMHHL HTN------- ---------- ----DVDADP DQK-AH-GVL R
CR        Aureococcus              LQEHVVLHHM HTG------- ---------- ----DVEYDP DAQ-LA-PVM R
CR        Phaeodactylum            QEQH-WTHHA YTN------- ---------- ----HAEMDP DSFGAE-PML L
CR        Paramecium               RRSHNFGHHS CVN------- ---------- ----HLELDR AFDTTF-PYI R
CR        Tetrahymena              RRSHNYGHHG CVN------- ---------- ----HYELDR AFDTTF-PLL R
Greens    Chlamydomonas            RYHHQVSHHI HCN------- ---------- ----DDALDE DVFSAF-PML R
Greens    Volvox                   RYHHQVSHHV HCN------- ---------- ----DDAFDE DVFSAF-PFL R
Greens    Ostreococcus             RYHHQVSHHI HCN------- ---------- ----DNAMDQ DVYTAM-PLL R
Greens    Ostreococcus             RYHHMVSHHS YCN------- ---------- ----DADLDQ DVYTAL-PLL R
          Dictyostelium            CHQHVIGHHL YTN------- ---------- ----VRNADP DLGQGE-IDF R
          Porphyra                 RNQHVIGHHV YTN------- ---------- ----VMGADP DLPVALSGDP R
          Synechocystis            RYRHNYLHHT YTN------- ---------- ----ILGHDV EIH-GD-GAV R
          Acaryochloris            KFRHNQLHHI YTN------- ---------- ----IEGYDN EIE-GD-GVV R
```

Supplementary Fig. 14
Fatty acid desaturase (continued)

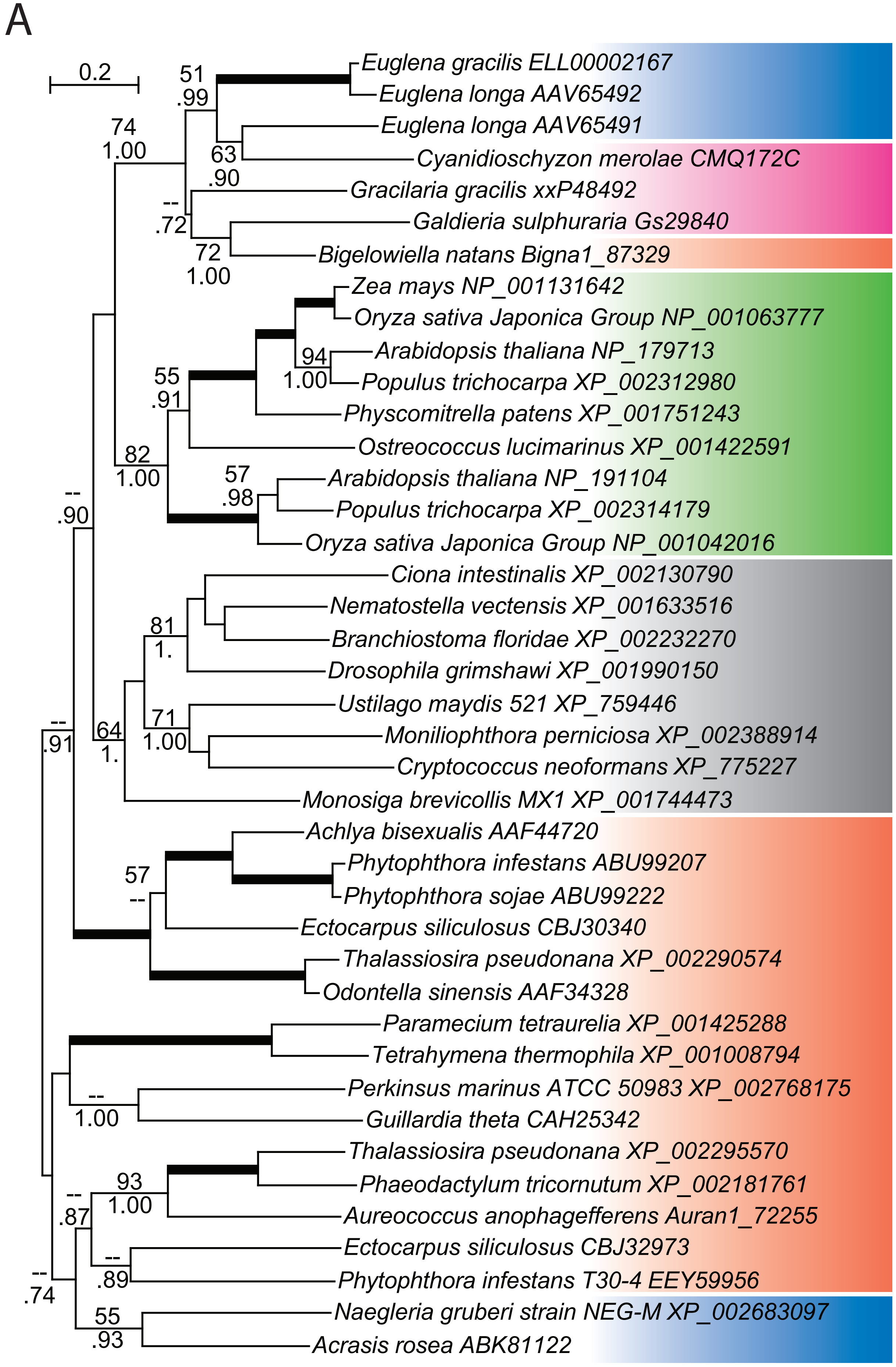

Sup. Fig. 15 Triose-phosphate isomerase

B

```
                             150                    172
         Euglena gracilis  WVILGHSERR -SLPEIKETD ETVATK
         Euglena longa 1   WVILGHSERR -SLPEIMETD ATVATK
         Euglena longa 2   WVILGHSERR -HLPELKESD ETVAIK
CR+Red   Bigelowiella      WVILGHSERR -HLPEIKETD EVIAKK
CR+Red   Galdieria         WVILGHSERR -HLPELQETD ETIAQK
CR+Red   Cyanidioschyzon   HVILGHSERR -HLPEIRESD DVIGRK
CR+Red   Gracilaria        WVILGHSERR -HIAQLKESD HTIAMK
Greens   Arabidopsis       WVILGHSERR -HV--IGEKD EFIGKK
Greens   Physcomitrella    WVIQGHSERR -HV--IGESN ETIGKK
Greens   Ostreococcus      WVILGHSERR -NL--FNESN EFVGKK
unikonts Cryptococcus      WVILGHSERR -SL--FGDTD KLVADK
unikonts Monosiga          WVILGHSERR -SI--FGESN ELIGEK
unikonts Nematostella      WVIIGHSERR -NI--FGEKD ELIGEK
unikonts Drosophila        WVILGHSERR -AI--FNESD ELIAEK
Exca.    Naegleria         STLLGHSERR -HV--FGESN TVIAKK
Exca.    Acrasis           YAIVGHSERR -SL--YHETD EVAAHK
CR       Tetrahymena       WAILGHSERR -QY--YGETN EVVANK
CR       Paramecium        WVILGHSERR -QY--NGETN EIVGKK
CR       Perkinsus         WTILGHSERR -HL--FNETN EDLAAK
CR       Guillardia        WVILGHSERR -EY--FKEDN EMLRQK
CR       Aureococcus       WVIVGHSERR -TN--QKESS ALVATK
CR       Phytoph. inf. 1   WTITGHSERR -AY--YNETD EIVAKK
CR       Phaeodactylum     WVIIGHSERR EGFEMPGETP DLCAKK
CR       Thalassiosira 1   WVILGHSERR EGFGMAGEDS ELVAKK
CR       Ectocarpus 1      WVITGHSERR VGFGCAGESS DLIAEK
CR       Odontella         WVVIGHSERR -GK---GEAD EEVARK
CR       Thalassiosira 2   WAIVGHSERR -GK---GEAD EEIAKK
CR       Ectocarpus 2      WCIIGHSERR -QK---GETN EECAVK
CR       Achlya            YTIIGHSERR -QK---GETN EEVASK
CR       Phytoph. inf. 2   YTLVGHSERR -EK---GETN EVVAKK
CR       Phytoph. soj.     YTLVGHSERR -EK---GETN EVVAKK
```

Supplementary Fig. 15
Triose-phosphate isomerase (continued)
